# Understanding the role of imidazolium-based ionic liquids in the electrochemical CO_2_ reduction reaction

**DOI:** 10.1038/s42004-023-00875-9

**Published:** 2023-04-29

**Authors:** Alessia Fortunati, Francesca Risplendi, Michele Re Fiorentin, Giancarlo Cicero, Emmanuele Parisi, Micaela Castellino, Elena Simone, Boyan Iliev, Thomas J. S. Schubert, Nunzio Russo, Simelys Hernández

**Affiliations:** 1 Department of Applied Science and Technology, Politecnico di Torino, Corso Duca degli Abruzzi 24, 10129 Turin, Italy; 2 Iolitec Ionic Liquids Technologies GmbH, Im Zukunftspark 9, 74076 Heilbronn, Germany

**Keywords:** Electrocatalysis, Electrocatalysis, Carbon capture and storage, Atomistic models, Chemical engineering

## Abstract

The development of efficient CO_2_ capture and utilization technologies driven by renewable energy sources is mandatory to reduce the impact of climate change. Herein, seven imidazolium-based ionic liquids (ILs) with different anions and cations were tested as catholytes for the CO_2_ electrocatalytic reduction to CO over Ag electrode. Relevant activity and stability, but different selectivities for CO_2_ reduction or the side H_2_ evolution were observed. Density functional theory results show that depending on the IL anions the CO_2_ is captured or converted. Acetate anions (being strong Lewis bases) enhance CO_2_ capture and H_2_ evolution, while fluorinated anions (being weaker Lewis bases) favour the CO_2_ electroreduction. Differently from the hydrolytically unstable 1-butyl-3-methylimidazolium tetrafluoroborate, 1-Butyl-3-Methylimidazolium Triflate was the most promising IL, showing the highest Faradaic efficiency to CO (>95%), and up to 8 h of stable operation at high current rates (−20 mA & −60 mA), which opens the way for a prospective process scale-up.

## Abstract (web summary)

Room-temperature ionic liquids are increasingly investigated as electrolytes for electrocatalytic CO_2_ reduction thanks to their high intrinsic ionic conductivities, wide electrochemical potential windows, and high CO_2_ absorption capacities and solubilities. Here, seven imidazolium-based ionic liquids are investigated as electrolytes for the electrochemical conversion of CO_2_ to CO using a silver foil cathode, with their stability, co-catalytic effects, and varying selectivities elucidated.

## Introduction

The emission of carbon dioxide is one of the problems receiving the most attention worldwide since it is one of the main causes of the greenhouse effect and the climate change, which threatens not only national economies but also the global environment^1–3^. Currently, three alternatives to address this problem are being examined: the first one is based on CO_2_ emissions reduction by using less carbon-rich forms of fuel for energy production and increase of renewable energies use^4,5^; the second one, concerns the development of new systems for CO_2_ capture and storage^6–8^; and the last one is related to the use of CO_2_ as feedstock for the synthesis of useful chemicals or fuels^9–12^. In this work, the electrochemical (EC) CO_2_ reduction reaction (CO_2_RR) aided by ionic liquid (IL)-based electrolytes is analyzed for the selective production of CO, which can be exploited along or in mixture with H_2_ (as Syngas) to be used as fuel or as intermediate for industrial processes (e.g., Fischer Tropsch^13^, hydroformylation reaction^14^, methanol synthesis^15^). This is not an easy task because CO_2_ is a very stable gaseous linear molecule; thus, due to the carbon atom sp hybridization and delocalized electrons in π-orbitals, it is very difficult to activate and reduce. For this reason, both the use of a new-designed electrocatalyst (e.g., based on Cu, Zn, Ag)^16–21^ in conjunction with electrolytes optimization are two crucial strategies under investigation in the research community.

Regarding the latter, on which this work is focused, room-temperature ionic liquids (RTILs) are being increasingly used as electrolytes for EC CO_2_RR thanks to their good intrinsic ionic conductivity, wide electrochemical potential windows and high CO_2_ absorption capacity and solubility^16,22^, with respect to aqueous-based electrolytes. Generally, RTILs have been coupled to metallic electrodes for the CO_2_RR (i.e. Ag, Au, Pt), but the best onset potential and current density values were reached with silver electrodes^23^. The most common ionic liquids used for the CO_2_RR contain imidazolium-based cations like 1-butyl-3-methylimidazolium [BMIM]^+^ and 1-ethyl-3-methylimidazolium [EMIM]^+^, with different anions (see Table S1 in the supporting information, SI). At the same time, based on the different chemical nature of the anion in the RTIL, the interaction of the cation with the CO_2_ and the electrode surface can be affected. Imidazolium-halide-based ionic liquids (e.g. containing [BF_4_]^−^, [PF_6_]^−^) are of large interest due to the stability of the cation ring and the anion hygroscopicity^24^. But, unfortunately, RTILs with fluorine-based anions are subject of debates about their potential decomposition into the toxic hydrofluoric acid (HF), which discourages their use as green electrolytes^25,26^. Another advantage of RTILs is their ability to stabilize EC CO_2_RR reaction intermediates, such as the CO_2_^•-^ anion radical that is the rate-determining step (RDS) of the reaction at high current densities^27^. For instance, Motobayashi et al.^28^ observed RTIL cations stabilizing the CO_2_^•-^ radical intermediate by using surface-enhanced infrared absorption spectroscopy (SEIRAS). Ratschmeier et al.^29^ also demonstrated the CO formation via an electrostatically stabilized CO_2_^•-^ radical by the RTILs cation by using in operando IR absorption spectroscopy and in situ sum-frequency generation spectroscopy. Thus, RTILs play a fundamental role as co-catalysts lowering the EC CO_2_RR overpotential^13,30,31^. On the other hand, the nature of anion also significantly affects the CO_2_ electroreduction performance: as an example, Golru et al.^32^ demonstrated that more hydrophilic anions can let more water molecules accumulate at the electrode surface and consequently enhance the side H_2_ evolution reaction (HER).

Based on this background, herein, seven imidazolium-based ILs (with and without fluorinated anions) were investigated as electrolytes for the EC conversion of CO_2_ to CO by using an Ag foil as cathode. They were chosen because of their higher CO_2_ capture ability than aqueous electrolytes. Since pure ILs have a high viscosity, which directly affects their conductivity and transport properties, an organic solvent was used as dilutant. The stability and co-catalytic effect of the imidazolium-based salts were investigated from the experimental and theoretical points of view. The catholyte properties such as conductivity and viscosity have been studied in detail to identify the best conditions for the CO_2_RR electrocatalytic tests. The role of the anion and the cation were studied and correlated to a change in the products selectivity. We experimentally and theoretically demonstrate for the first time that the role of the IL anions is to tune the ratio between the cations [EMIM]^+^ or [BMIM]^+^ and the carbene species (EMIM: or BMIM:) in the electrolyte and, consequently, the CO_2_ capture and electrochemical conversion features of the ILs. When using acetate as anion, the dissolved CO_2_ is chemically and irreversible absorbed by the carbene and, thus, not available for the EC CO_2_RR. Moreover, the proton exchange equilibrium between the [EMIM]^+^ and the acetate (being a strong Lewis base), greatly favors the formation of the carbene EMIM: that leads to a covering of the Ag electrode surface. Instead, by using fluorinated anions (weak Lewis bases), a greater concentration of IL-cations favors the EC CO_2_RR, by stabilizing the adsorbed CO_2_ (i.e., CO_2_* formation) at the Ag surface, as well as by decreasing the overpotential of all the reaction intermediates of the CO_2_-to-CO EC conversion process. Finally, durability tests of the most promising catholyte based on [BMIM][SO_3_CF_3_] were carried out to evaluate its long-term performance for a future improvement and scale up of the system.

## Results and discussion

### Catholytes properties: conductivity and viscosity

The ILs that were investigated as electrolytes for the CO_2_ electroreduction reaction are listed in Table 1. Because of their high viscosity, pure ILs present very low conductivity. For this experimental work, making the catholytes as conductive as possible was fundamental to lower the total cell overpotential, considering the distance between the electrodes and the use of a bipolar membrane separating the two chambers. Thus, to decrease the electrolyte resistance, diluting the ILs in a suitable solvent was essential.

**Table 1:** Ionic liquids investigated.

Ionic Liquid	Abbreviation	Cation	Anion
**1-Butyl-3-Methylimidazolium Tetrafluoroborate**	[BMIM][BF_4_]		
**1-Butyl-3-Methylimidazolium Acetate**	[BMIM][CO_2_CH_3_]		
**1-Butyl-3-Methylimidazolium Triflate**	[BMIM][SO_3_CF_3_]		
**1-Butyl-3-Methylimidazolium Trifluoroacetate**	[BMIM][CO_2_CF_3_]		
**1-Ethyl-3-Methylimidazolium Acetate**	[EMIM][CO_2_CH_3_]		
**1-Ethyl-3-Methylimidazolium Triflate**	[EMIM][SO_3_CF_3_]		
**1-Butyl-2,3-Dimethylimidazolium Acetate**^a^	-		

^a^This IL was used only to confirm the proposed mechanism of reaction of [BMIM] [CO_2_CH_3_] and [EMIM][CO_2_CH_3_].

**Fig. 1:**
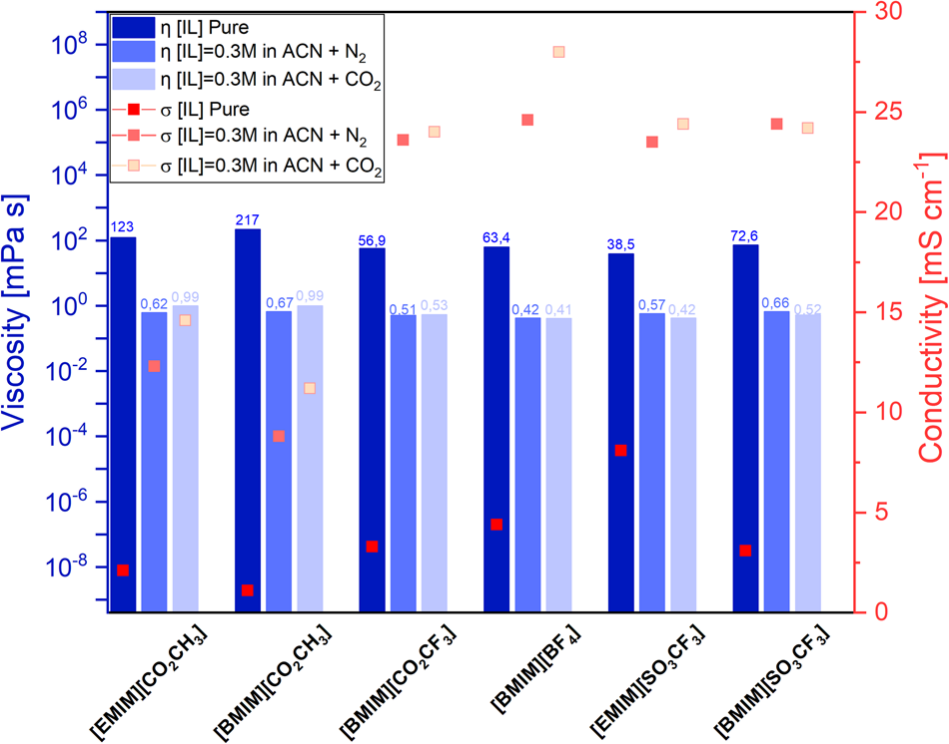
Conductivity and viscosity properties of the ILs-based electrolytes. Comparison between pure ionic liquids and their solutions in acetonitrile both in N_2_ and CO_2_ saturated atmosphere. Conductivity and viscosity results were reported in red scatter and blue bar graphs, respectively.

Figure 1 shows the viscosity (columns) and conductivity (squares) values of pure ionic liquids and compares them with their respective viscosity (light blue columns) and conductivity (pink squares) in a 0.3 M solution in acetonitrile. A large decrease in viscosity and a sharp increase in conductivity can be noticed when switching from the pure ILs to their respective solution. Pure [EMIM][CO_2_CH_3_] and [BMIM][CO_2_CH_3_] present the highest viscosities, *η* = 123 mPa s and *η* = 217 mPa s, respectively. The lower conductivity of the two acetate salts than of the other ILs probably is due to hydrogen bonds that the acetate anion can form with other anion-cation specie in solution or due to the strong cation-anion interaction that cause a low IL ionicity^33,34^. The viscosity is similar for all the IL solutions in ACN and equal to ~0.4/0.7 mPa s. This can be linked to the fact that all the solutions are very diluted ([IL] = 0.3 M). The properties of the final solutions in terms of viscosity almost reflect the properties of the pure solvent. Indeed, the pure acetonitrile has a viscosity equal to *η* = 0.15 mPa s. From the point of view of conductivity, ACN shows a very low value, *σ* = 5 µs cm^−1^, while pure ILs present higher values in a range between σ_[BMIM][CO2CH3]_ = 1.1 mS cm^−1^ and σ_[EMIM][SO3CF3]_ = 8.1 mS cm^−1^. The dissolution of ILs in acetonitrile permit to reach higher values of conductivity than such of the pure solvent, ranging from σ_[BMIM][CO2CH3]_ = 8.8 mS cm^−1^ to σ_[BMIM][BF4]_ = 24.6 mS cm^−1^. The trend is the same for the 0.3 M IL solutions saturated either in N_2_ or in CO_2_. Small variations of the viscosity are observed, in the order of a maximum of 0.3 mPa s, when passing from N_2_ to CO_2_ saturated solutions. However, more notable variations were appreciated for the conductivity values, which increased in the order of a maximum of 3 mS cm^−1^ in CO_2_ with respect to N_2_, especially for acetate-containing ILs and [BMIM][BF_4_].

### Electrochemical stability of ILs at negative potentials

The ILs stability is of paramount importance in selecting the appropriate one for a specific application. The electrochemical stability of such substances is usually expressed by the width of the electrochemical window, that refers to the difference in voltage between the oxidation and reduction potentials of the ionic liquid^35^. The limit potential to evaluate such window is an arbitrary cut-off absolute value of the current density (I_Cut-Off_), which is commonly set between 0.01 and 1 mA cm^−2,^^36–39^. Many other parameters might influence the width of the potential window, being the most important one the type of working electrode and in a lesser extent, the scan rate. Generally, the most used working electrodes are made of platinum, glassy carbon (GC) and tungsten^35^. Besides the advantages of acetonitrile when used as solvent of the imidazolium salts, due to the increment of conductivity because of a decrease in the viscosity of the ionic liquid, McEwen et al.^40^ demonstrated that acetonitrile along with butylene carbonate provide a wide window of stability of about 4.0 V vs Ag_wire_. In the first case, because of the highly conductive characteristic and in the second, due to the substitution of the imidazolium cation in the C2 position with an alkyl group that increases the steric hindrance. In this work, a I_Cut-Off_ of 1 mA cm^−2^ was selected. To calculate the stability window, it was chosen to perform the tests with the same two compartment cell used for the CO_2_RR and with the same WE, CE and membrane, to have similar overpotentials to those encountered during the electrocatalytic tests. The experimental conditions used to measure the stability window are summarized in Table S2 (SI). Cyclic voltammetry (CV) measurements were recorded in the IL-based solutions in ACN saturated with an inert gas (N_2_), to study the contribution to the current variation of the electrolyte under test. Measurements in a CO_2_ saturated atmosphere were not performed to avoid the contributions to the current variation made by the CO_2_RR. Moreover, due to the redox potential of Ag, which was used as working electrode, the oxidation region could not be investigated to avoid the transformation of Ag(s) into Ag^+^ or Ag^2+^ when applying a positive potential. On the other hand, only the range of negative potentials is of interest for studying the behavior of the electrolytes in CO_2_ reduction condition. In terms of the potential reached to obtain the fixed I_Cut-Off_, the trend observed is the following (see Fig. S4, SI): [EMIM][SO_3_CF_3_] = −2000 mV > [BMIM][CO_2_CF_3_] = −1995 mV > [BMIM][BF_4_] = [BMIM][SO_3_CF_3_] = −1900 mV > [BMIM][CO_2_CH_3_] = −1887 mV > [EMIM][CO_2_CH_3_] = −1860 mV (where all the potential values are referenced to the Ag/AgCl electrode). According to Kazameiabnavi et al.^41^, the electrochemical stability window of these type of ionic liquids is limited by the reduction potential of the cations and the oxidation potential of the anions. It is evident from the above-described results, that the change in the cathodic potential is not so significative. This could be explained because the reduction potential of 1-alkyl-3-methilimidazolium cations remains almost constant despite the length of the alkyl group, since they contain identical imidazolium ring structures. Thus, the slight differences could be related to the different conductivity values of the solutions. In fact, considering the four ionic liquids with the same cation [BMIM]^+^, the lowest stability window was obtained for the [BMIM][CO_2_CH_3_], which has the lowest conductivity and highest viscosity of the here studied ILs (as reported in Fig. 1). Likewise, considering the two ionic liquids with the same [EMIM]^+^ cation, a higher potential stability window was obtained for the [EMIM][SO_3_CF_3_] than for [EMIM][CO_2_CH_3_], because of the higher conductivity of the triflate-based solution, while the latter has similar characteristics in terms of viscosity and conductivity to [BMIM][CO_2_CH_3_]. In general, ILs with fluorinated anion are more conductive, less viscous, and therefore have a slightly wider window of cathodic electrochemical stability. Since these measurements were done in the absence of CO_2_, the cathodic current increase can be attributed both to the HER and to the decomposition of the catholyte itself. To experimentally confirm the extend of the H_2_ production, crono-potentiometry (CP) studies at −20 mA cm^−2^ with N_2_-saturated solutions of [BMIM][SO_3_CF_3_] and [BMIM][CO_2_CH_3_] were performed and a FE_H2_ = 13% and FE_H2_ = 15% were obtained, respectively (Fig. S5 and Fig. S6, SI). Moreover, a color variation of the catholyte solution from transparent to dark yellow was noticed (see Figs. S5A, B, S6A, B). Considering both that the faradaic efficiency values for the HER were far from 100% and that the catholyte changed color, it was observed that in the absence of other Faradaic reactions (being in absence of CO_2_RR) the IL decomposition is highly probable. As demonstrated by De Vos et al.^35^ through NMR analyses, alteration of imidazolium-based ILs concern only the cation, in particular at the C2 position with the loss of aromaticity and the possible formation of radicals, dimers, disproportionation reaction and cagelike structures.

### Equilibrium CO_2_-Ag-ILs: comparison based on different IL counterions

Before voltammetry studies were performed, the catholyte was purged with an inert gas (N_2_) to remove oxygen and other gaseous impurities, followed by saturation with CO_2_, at room temperature. CO_2_ and electrolyte interactions that can occur during the saturation phase, before the electrochemical test, will be discussed in following.

It is widely known in literature that some RTILs have a high ability to capture CO_2_^42^. This peculiarity is found in ILs with carboxylate-functionalized anions, e.g. the acetate anion in this work, since they are able to chemisorb the CO_2_ molecule. Maginn et al.^43^ classified CO_2_-[BMIM][CO_2_CH_3_] a case with strong intermolecular interactions and complex formation. Chin et al.^44^ showed a mechanism where CO_2_ is captured as a bicarbonate group on this IL. Blath et al.^45^ performed infrared spectroscopy on [EMIM][CO_2_CH_3_] to study the CO_2_-IL interaction. They compared IR-spectra of the untreated IL in its pure condition with the CO_2_-treated one. In the latter spectrum, they found a peak at 2345 cm^−1^ that represents the CO_2_ dissolved and chemical unbounded, and a second peak at 1663 cm^−1^ that Tommasi et al.^46^ correlated with the carboxylate formation.

**Fig. 2:**
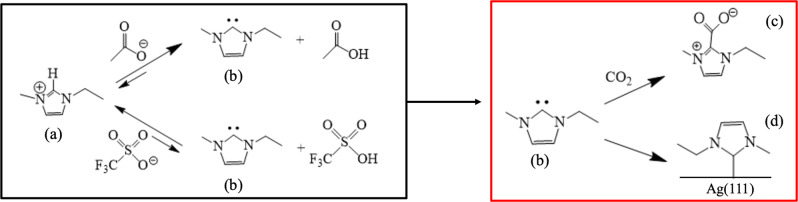
Chemical equilibrium of imidazolium in the presence of different anions: a non-fluorinated ([CO_2_CH_3_]^−^) and a fluorinated one ([SO_3_CF_3_]^−^). Since this reaction mechanism foresees only the involvement of the imidazolium ring, and the alkyl chain does not play a key role, it is assumed that the same reactions occur for the [BMIM]^+^ cation. **a** Represents the cation, [EMIM]^+^; (**b**) is the carbene, EMIM:, (**c**) is the carboxylated cation, and (**d**) is the cation chemisorbed in the Ag surface, [EMIM]*.

The mechanism behind the CO_2_ capture by [CO_2_CH_3_]^−^-containing ILs can be described as follows: according to aqueous solutions equilibrium, acetic acid is a weak acid^47^, and consequently acetate is a strong conjugated base. According to Watanabe et al.^33^, strong ILs anion can be considered as Lewis bases. Therefore, the proton exchange equilibrium between the [EMIM]^+^ and the acetate shown in Fig. 2 (left), greatly favors the formation of the carbene species, namely EMIM: (B in Fig. 2), which is a Lewis base. The formed carbene species will then react with the dissolved CO_2_ (a Lewis acid), forming carboxylate species (C in Fig. 2, right)^48^. Cabaço et al.^49^ and Chiarotto et al.^50^ also experimentally demonstrated that in the case of acetate-based ILs, carbene species are formed from the [BMIM]^+^ cation, which reacts with CO_2_ to form [BMIM]^−^CO_2_, whereas the proton released by the ring interacts with the anion to produce acetic acid.

By replacing the [CO_2_CH_3_]^−^ anion with the [CO_2_CF_3_]^−^, the behavior of the IL towards the CO_2_ molecule radically changes. Considering the lower basic strength of [CO_2_CF_3_]^−^ in comparison to the [CO_2_CH_3_]^−^ anion in aqueous equilibrium^51^, the force of the base to tear the H in C2 decreases and the formation of the carbene is strongly reduced, leading to less carboxylate development. In this case, the capability of ILs to capture CO_2_ is negligible and, consequently, CO_2_ is only dissolved in the solution via weak van der Waals interactions. This occurs also in the case of other fluorinated anions such as [SO_3_CF_3_]^−^ and [BF_4_]^52,53^. Therefore, the quantity of deprotonated cations (or carbene species) in the system tightly depends on the nature of the anion. The stronger the conjugate-base anion, the greater the tendency to deprotonate the cation. Based on this, we envisage that the role of anions is then to tune the ratio between [EMIM]^+^/EMIM: in the solution and thus the CO_2_ capturing feature of the IL.

It is known that Ag, the cathode employed in this study, also behaves as a Lewis acid, and, as such, it can react with the carbene species present in the electrolyte solution. To characterize the reactivity of carbene with respect to the CO_2_ and Ag surface, we have first studied the possible reactions by means of DFT. The Ag(111) facet was used for the study being the most representative of the electrode surface, as confirmed by XRD analysis (See Fig. S7, SI). The reactions can be studied by computing the binding energies (BE) of carbene and CO_2_ and carbene and Ag(111), referenced to the isolated molecules and surface. The CO_2_ binding energy (BE) is negative in both cases: BE_CO2-EMIM:_ = −0.60 eV and BE_CO2-BMIM:_ = −0.67 eV for EMIM: and BMIM:, respectively. The binding energy of EMIM: on the Ag(111) surface results to be BE_Ag(111)-EMIM:_ = −1.86 eV. Moreover, NEB calculations prove that all these reactions are barrierless and spontaneously occur (see results in Fig. S8, SI). Since BE_Ag(111)-EMIM:_ < BE_CO2-EMIM:_, we can argue that carbenes preferably bind to the Ag electrode surface and the remaining carbene species in the solution capture the dissolved CO_2_ via chemical reaction (i.e. chemical absorption). These results demonstrate that EMIM: occupies the active sites of the Ag catalyst surface. It has been shown that entropic and enthalpic corrections do not change these conclusions^54^. To sum up, in the case of ILs with acetate anions, a high quantity of neutral carbenes (EMIM: or BMIM:) are present in solution, which react with the CO_2_ molecules and with the electrode surface. In the case of ILs with fluorinated anions, carbenes are not widely formed, thus, the imidazolium remains the dominant species in the electrolyte and CO_2_ remains dissolved in the solution as a neutral specie. [EMIM]^+^ and [BMIM]^+^ will then be attracted to the electrode interface, under the action of the electric field, participating in the CO_2_RR process.

## Electrochemical study of CO_2_RR in IL-based solutions

The CO_2_ reduction activity of Ag in presence of different ILs was studied by CV, linear sweep voltammetries (LSV) and CPs.

### Cyclic voltammetry study in N_2_ and CO_2_

The results of CVs recorded after the catholyte outgassing in N_2_ and saturation in CO_2_ are reported in Fig. S9 (SI). The onset values (i.e. potential applied to start a Faradaic process) obtained with all ionic liquids in N_2_- and CO_2_-saturated atmospheres show that, in general, reduction processes in the presence of CO_2_ take place at more positive applied potentials than in N_2_, mirroring the electrolyte conductivity trends discussed in the previous sections. CV curves in presence of CO_2_ for the two ILs with acetate, [BMIM]/[EMIM][CO_2_CH_3_], lie at lower potentials (in absolute value) than in the case of fluorinated ILs. This can be related to the electrolyte being richer in protons, hence favoring any reduction process. Acetate-containing ILs present a larger number of available protons for two fundamental reasons. Firstly, they have a higher water content than the fluorinated ones, being more hydrophilic. Secondly, as discussed, the acetate anions favor the production of carbene molecules by deprotonating [EMIM]^+^ or [BMIM]^+^ and turning into acetic acid. Water and CH_3_COOH can then serve as proton reservoirs from which reduction reactions can proceed already at lower cathodic potentials than in the case of fluorinated ILs, where the few available protons originate from the membrane, due to anolyte crossover or, possibly, some traces of water in the non-perfectly anhydrous ACN. This suggests that, in principle, acetate-containing ILs could reduce CO_2_ more easily than fluorinated ones, but also the high availability of protons can favor the HER. Hence, it is the inline gas analysis, performed during the CPs and discussed in section 3.3.3, that allows to identify the species that are reduced.

### Linear sweep voltammetry study in CO_2_

The LSV curves performed in CO_2_ saturated atmosphere with the six investigated ILs are reported in Figs. S10, S14 (SI). The comparison between the pure ACN (black line) and the 0.3 M ILs solutions (colored lines) shows how the presence of the ILs positively influences the onset potential of the CO_2_RR, i.e. the potential at which the reaction starts to occur. Regardless of the nature of the anion and cation, the LSV curves with all ILs report much higher currents, in the same potential range, than pure ACN. From these analyzes it can be concluded that each solution of [IL] = 0.3 M was more active in the CO_2_ reduction processes than pure ACN. Figure S10A compares the four different ILs with the [BMIM]^+^ cation, to evaluate the sole influence of the anion in the Ag electroactivity. Figure S10B compares two different [EMIM]-based ILs. The specific ILs activity, the respective curves position, and the total current densities achieved were a function of the proton concentration in the solution and the viscosity/conductivity properties. Acetate-containing ILs show a lower onset potential value in accordance with what was highlighted and discussed for the CVs. CP studies were conducted at different current values (see section 3.3.3) to investigate the real reduction process in progress, i.e. to confirm whether it was HER or CO_2_RR.

### Potentiometry study in CO_2_

CPs were recorded to assess the stability and selectivity of the EC CO_2_RR reaction at different fixed currents (*I* = −20/−10/−5 mA). To distinguish which reaction is taking place, the gas products from the cathode chamber were analyzed through an inline µ-GC for all the duration of the test, as described in the Experimental section. The CP trends with the different ILs electrolytes are reported in Fig. S11 (SI) at the highest current value. In general, the cathodic potential during the CPs with [BMIM][BF_4_] electrolyte is stabilized at a lower value (e.g., −1.65 V vs Ag/AgCl at −20 mA) than with the other ILs due to its high conductivity (i.e. 28 mS cm^−1^, see Fig. 1). [BMIM][SO_3_CF_3_], [EMIM][SO_3_CF_3_] and [BMIM][CO_2_CF_3_] have about the same conductivity value (~24 mS cm^−1^) and, their CPs stabilized at nearly the same potential (e.g. −1.8 V vs Ag/AgCl at −20 mA). [BMIM][CO_2_CH_3_] has a much lower conductivity value (about 11 mS cm^−1^) and its CP stabilized at a higher cathodic potential (e.g., −2.1 V vs Ag/AgCl at −20 mA). Hence, a correlation between conductivity viscosity and cathodic potentials during CPs was clearly observed.

**Fig. 3:**
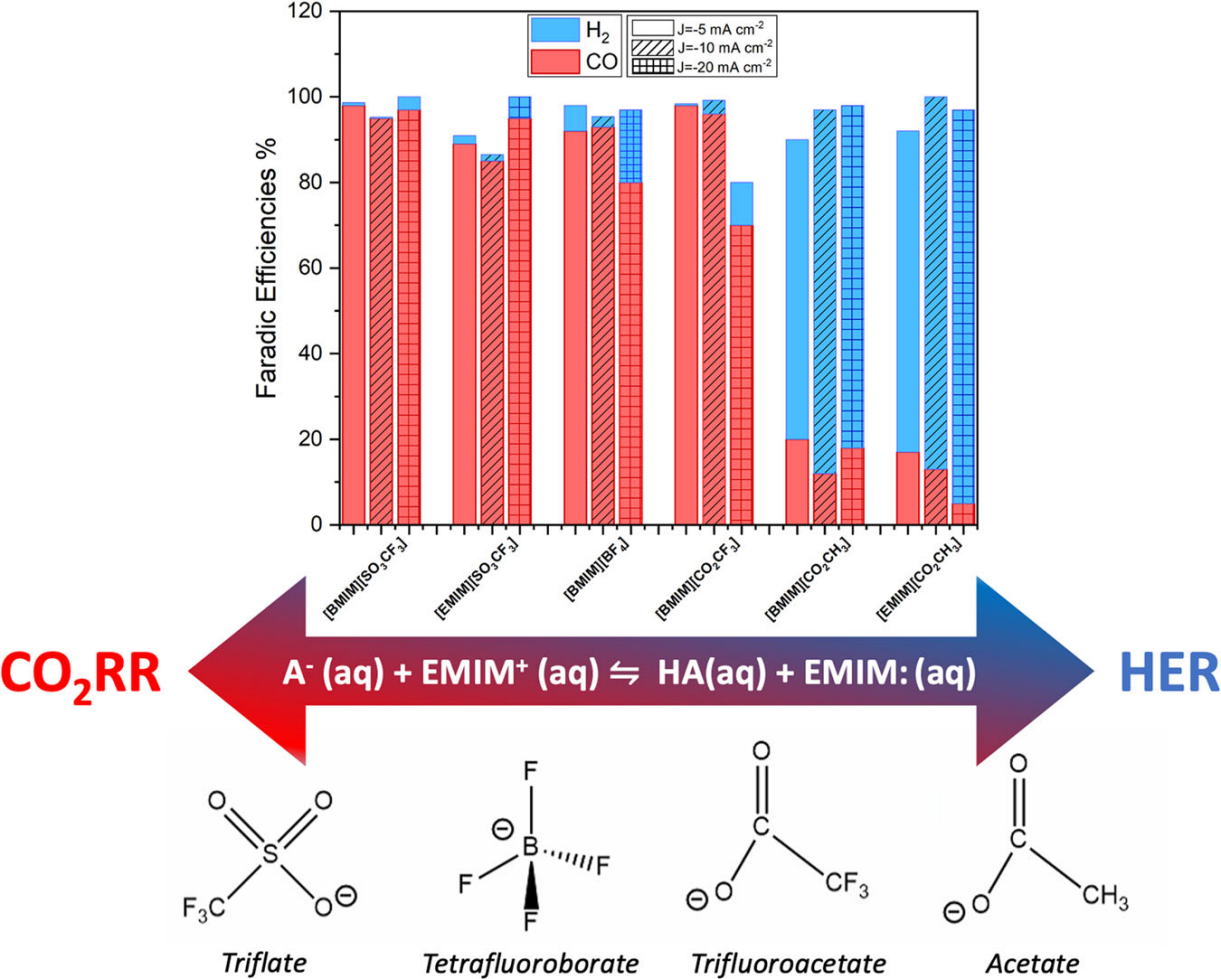
Faradic efficiency values of the gaseous products generated during the EC CO_2_RR. Chronopotentiometries (CPs) were conducted at constant currents of I = −20 mA, I = −10 mA, I = −5 mA, with the different ILs. Set-up: H-cell; WE: Ag foil; catholyte: 0.3 M IL in ACN; CE: Ni mesh; anolyte: 0.1 M KOH.

Concerning the selectivity towards the CO_2_ reduction, the results from the analysis of the gaseous products during the CPs were used to calculate the FE towards CO and H_2_, and the results are reported in Fig. 3. A change in the selectivity between the CO_2_RR to CO and the HER was observed by varying the type of ionic liquid. From the bar graph it can be appreciated that ILs with fluorinated anions have a greater tendency to favor the reduction reaction of CO_2_ to CO instead of favoring HER at all the studied current values. In particular, [BMIM][SO_3_CF_3_] maintains a FE_CO_ > 95% at any current density. For [EMIM][SO_3_CF_3_] there is a decrease in CO production, going from the maximum FE_CO_**~**95% at I = −20 mA to FE_CO_~85–90% for I = −10 mA and I = −5 mA, respectively. In the case of [BMIM][BF_4_] and [BMIM][CO_2_CF_3_] the highest selectivity for CO (FE_CO_ = 93% and 97%) was obtained at I = −10 mA and at I = −5 mA, respectively. At the same time, by using ILs with acetate anion, H_2_ was the major gaseous product obtained. By applying a current of I = −5 mA, both in the case of [BMIM][CO_2_CH_3_] and [EMIM][CO_2_CH_3_], the maximum production of CO is obtained, reaching FE_CO_ = 20% and 17%, respectively.

This is in accordance with what has been described in the reaction mechanism in Fig. 2: when using as catholyte an IL containing the [CO_2_CH_3_]^−^ anion, carbene molecules are formed which either chemisorb on the electrode surface or react with the CO_2_ in solution carboxylates. Either way, the electroreduction of dissolved CO_2_ is inhibited and hydrogen is the most abundant product. The low amount of CO collected when using the [CO_2_CH_3_]-containing ILs (i.e., FE_CO_ < 20%) is probably due to the reduction of not-bonded CO_2_ in solution that manages to reach the electrode surface. On the contrary, by using fluorinated ILs, there is a greater concentration of the cations ([EMIM]^+^ or [BMIM]^+^) that favors the CO_2_RR. Indeed, those positively charged species are attracted to the Ag electrode surface, under the action of the electric field, because of the negative applied potential at the cathode that induces the negative charging of the electrode. Based on Gang Li et al.^55^ results, due to electrostatic attractions, the negative WE is randomly covered by the positively charged cations of fluorinated IL in the innermost charged layer.

To sum up, in case of acetate-containing ILs, the chemisorbed carbene species at the Ag electrode surface (see section 3.3.4) make it slightly active for CO_2_RR (FE < 20%) and very active for HER. So, the catalyst has not been totally poisoned, losing its catalytic activity, but when covered by carbene it plays an inhibitory role not favoring the conversion of CO_2_, probably due to steric hindrance. The covering of the electrode by the carbene, together with the formation of the carboxylate, are two side reactions which disadvantage the electrochemical conversion of CO_2_. However, the different selectivity induced by different imidazolium-based ILs with different anions and the mechanism behind such behavior have not been previously investigated and, thus, it was in deep studied here by theoretical calculations as shown in the section 3.4. It is also worth highlighting that the here reported results evidenced a preferential selectivity for the CO_2_RR when using the [SO_3_CF_3_]^−^ anion against the most used [BF_4_]^−^.

### Analysis of Ag electrode surface after EC tests

The formation of carbenes and their chemisorption on the Ag surface is supported by the results of Raman and XPS characterizations of the electrode after testing it by a CP at −20 mA in [BMIM][CO_2_CH_3_] for 2 h. After the electrochemical test, the electrode was rinsed thoroughly with ACN before the Raman analysis, to remove all physisorbed molecules.

**Fig. 4:**
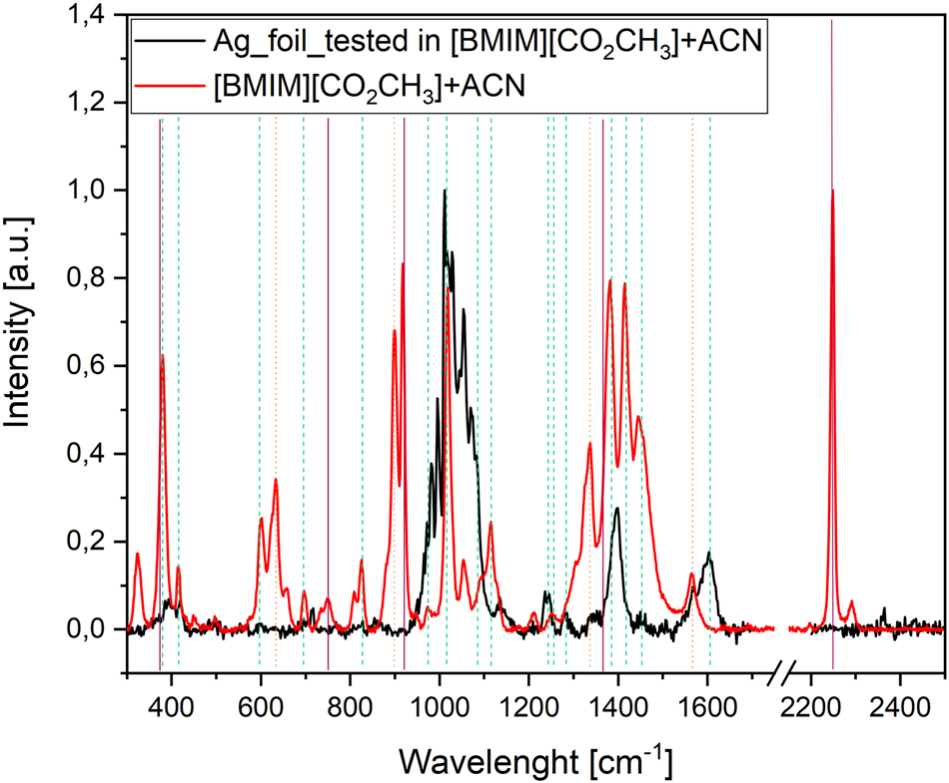
Raman spectra of the [BMIM][CO_2_CH_3_] electrolyte and working electrode after test. The electrolyte was composed by [BMIM][CO_2_CH_3_] in ACN (red line). The working electrode was after test (black line). Vertical lines mark intensity peaks related to acetate anion (dot orange line), BMIM cation (dashed green line) and finally ACN (solid purple line).

In Fig. 4, the Raman spectra of [BMIM][CO_2_CH_3_] diluted in ACN, and of the electrode after the test are shown. In the spectrum of the pure electrolyte, all peaks can be assigned to the vibrational modes (marked by the dotted vertical lines) of the anions and cations in the acetate-IL^56^ and of ACN^57^. Such assigned modes are reported in Table 2. In the spectrum of the electrode after test, the frequency range between 900 and 1100 cm^−1^ presents strong components attributed to vibrational modes of the Ag electrode, see Fig. S12B. In Fig. 4, despite the cleaning in ACN, the spectrum of the post-test electrode still shows peaks in correspondence to the peaks of the pure electrolyte attributed to the cation. In particular, the black spectrum shows peaks at 1600 cm^−1^, characteristic of the aromatic stretching mode ν_C=C_, at 1245 cm^−1^, of the skeleton stretching ν_C-C_ and at 1381 cm^−1^, characteristic of ν_C_Im-N_. It is crucial to notice that no peaks related to acetate anions or to acetonitrile are present.

**Table 2:** Raman vibrational mode peaks attribution regarding spectra in Fig. 4^82^.

[CO_2_CH_3_]^−^		[BMIM]^+^		ACN	
ν (cm^−1^)	mode	ν (cm^−1^)	mode	ν (cm^−1^)	mode
635	δ_OCO_ + ν_CC_	388	δ_C-Et-N_	380	δ_C-C≡N_
899	δ_OCO_ + ν_CC_	440	δ_NC_Im_	750	Octave
1334	ν_CO_	597	ν_Et-N +_ ν_Me-N_	920	ν_C-C_
1567	Anti ν_CO_	701	ν_Et-N +_ ν_Me-N_	1370	CH_3_ deform.
		830	ν_C-C-C but_	2200	ν_C≡N_
		957	ν_C_Et_		
		1019	ring deform.		
		1089	δ_H_Et_		
		1118	δ_H_Et_		
		1245	ν_C-C skel_		
		1295	Twist–(CH_2_)n		
		1381	δ_H_Et +_ ν_C_Im-N_		
		1417	δ_H_Me_		
		1451	δ_H_Et_		
		1600	ν_C=C ar_		

Consequently, the presence of adsorbed carbenes on the electrode surface can be deduced, since its Raman peaks appear in the spectrum of the cleaned post-test electrode, while no peaks of the acetate anion are present, suggesting that the remaining molecules on the surface are not in the cationic form. In the SI (Fig. S12), Raman spectra comparing each electrolyte components (a) and fresh silver electrode (B), before testing, are reported.

**Fig. 5:**
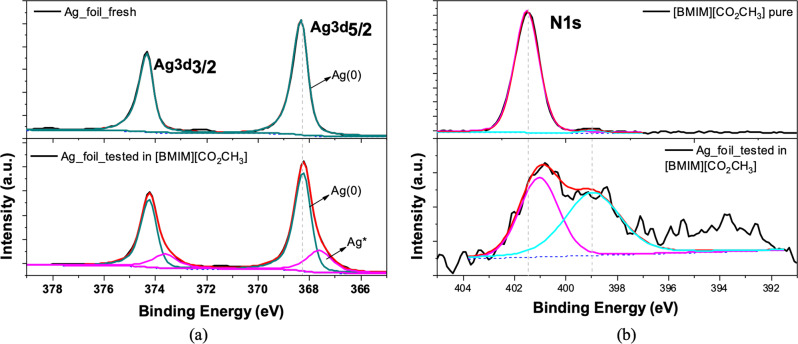
XPS spectra of the [BMIM][CO_2_CH_3_] electrolyte and working electrode after test. **a** Ag3d high resolution XPS spectra for the electrode before (upper panel) and after (lower panel) the electrochemical test. **b** N1s high resolution XPS spectra of the pure [BMIM][CO_2_CH_3_] IL (upper panel) and the tested electrode (lower panel).

The high-resolution XPS spectra of the Ag3d peaks of both the pristine (fresh) and the post-test electrode are reported in the upper and lower panels of Fig. 5a, respectively. The post-test electrode spectrum shows additional components at slightly lower binding energies than the Ag(0) peaks, which are consistent with the formation of a chemical bond with silver changing its oxidation state. This is confirmed by the concurrent analysis of the N1s spectra of the pure electrolyte (upper panel) and post-test electrode (lower panels) in Fig. 5b. In the upper panel, the pure electrolyte shows a single-component peak at 401.5 eV, given by the N atoms in the imidazole ring^58^. The peak in the spectrum of the post-test electrode shows two components, at 401.2 eV and 398.95 eV. The higher energy component is in agreement with the reported binding energies attributed in the literature to the N atoms in adsorbed carbene molecules^59–61^. The low-energy component is consistent with the binding energy of amines (N-H)^61,62^. Amine groups are absent from the pure electrolyte but can be easily produced by the decomposition of the IL during the prolonged electrochemical test.

The Ag electrode was also analyzed by FESEM and EDX analysis after the CP in [BMIM][CO_2_CH_3_]. Before these analyses, the electrode was rinsed several times with ACN to remove any residues and not chemisorbed species as confirmed by EDX analysis (see Table S3, SI). FESEM images of the fresh (Fig. S13A, SI) and post-tested and cleaned electrode (Fig. S13C, SI) evidence an increase of the roughness of the electrode surface. According to recent results from Jaramillo et al.^63^, this could be attributed to Ag reconstruction, which should be due to oxidation and reduction of the material during the electrochemical measurements or to Ostwald ripening processes.

Furthermore, to indirectly demonstrate the formation of the carbene according to the mechanism explained in the previous sections, the imidazole ring was methylated the C2 position by synthesizing the 1-butyl-2,3-dimethylimidazolium acetate. Thus, the more acidic C2 carbon in the imidazolium ring, which is the most proton-releasing, was capped by the methyl group and, therefore, it cannot be deprotonated. This would lead to an inability to form the carbene in C2 position. Thus, the 1-butyl-2,3-dimethylimidazolium acetate was diluted in acetonitrile and then tested in the H-cell for the EC CO_2_RR and the results are reported in the SI (Fig. S14). From the gaseous products trend, measured during a CP of one hour at I = −20 mA, it can be deduced that the production of CO was favored over the production of H_2_ (FE_CO_ = 68,7%; FE_H2_ = 24,4%). Thus, by using 1-butyl-2,3-dimethylimidazolium acetate, a larger amount of CO was collected with respect to [BMIM] and [EMIM] acetate ILs. This confirms that the acetate anion did not strip any hydrogen from the C2 position of the imidazole and no carbene was formed. However, comparing the performance of 1-butyl-2,3-dimethylimidazolium acetate with the fluorinated ionic liquids, the amount of CO produced was lower. Following the reaction mechanism of Feng et al.^64^, stabilization of the CO_2_RR reaction intermediates was found to be less effective when conducted by the hydrogens at C4 and C5, than by the hydrogen at C2.

The Raman spectra comparing the Ag electrode after the test and after testing and washing with ACN are shown in Fig. S15 (SI). The electrode after testing shows the characteristic peaks of the imidazole and acetate functional groups already assigned in Table 2 (see Fig. S15, purple line). Instead, the electrode after testing and washing with ACN shows the characteristic peaks of pure Ag (Fig. S15, black line), indicating that any organic compound remained chemisorbed on the electrode surface, which excludes the presence of carbene residues and, once again, confirms our previous results and discussion.

### DFT insight into the EC CO_2_RR mechanism over Ag surface

The role of the cation as co-catalyst in the electrochemical CO_2_ reduction was proved by DFT calculations.

**Fig. 6:**
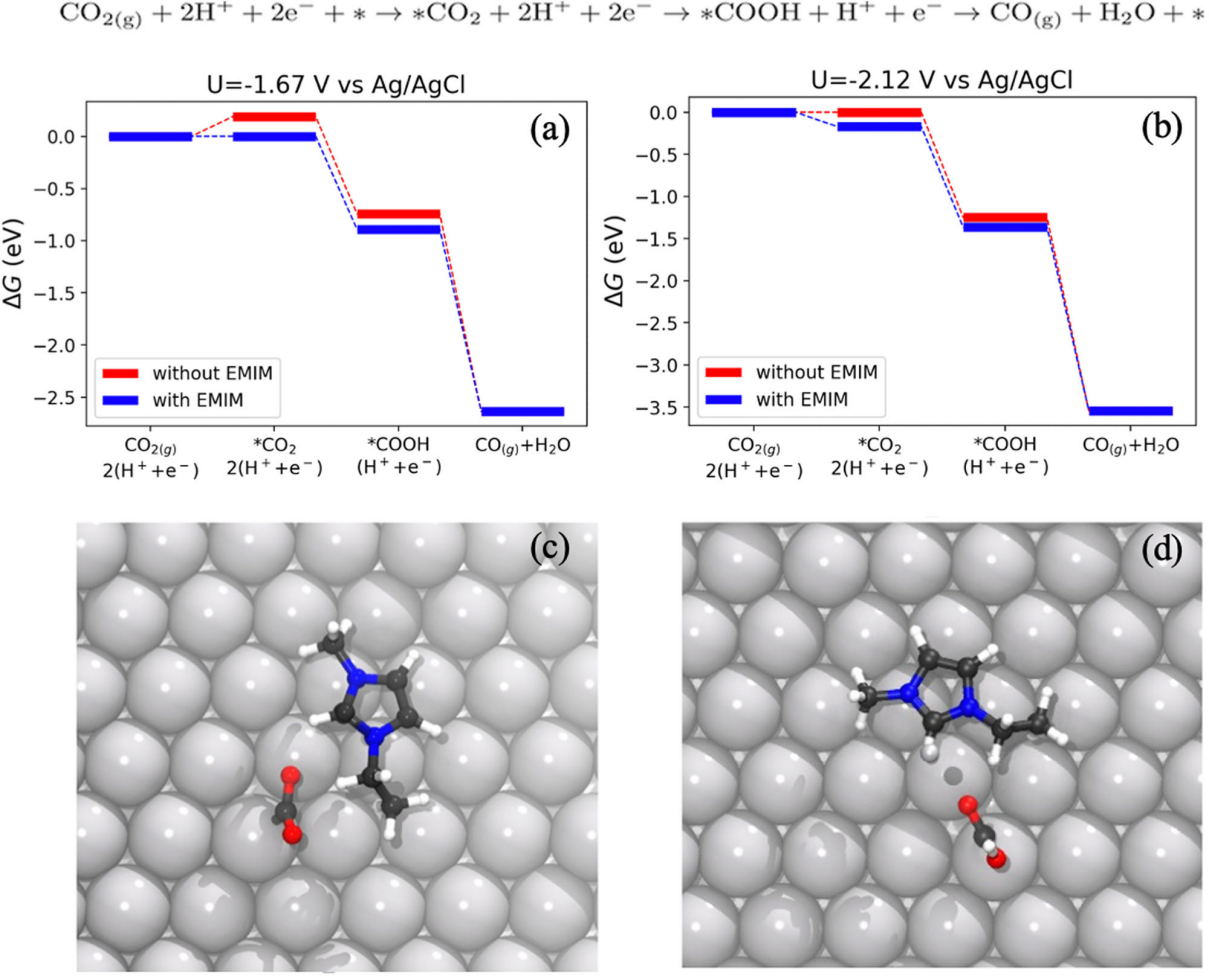
CO_2_RR reaction path to CO in acetonitrile. **a** − 1.67 V vs. Ag/AgCl and (**b**) −2.12 V vs. Ag/AgCl, on Ag(111) surface with and without [EMIM]^+^. The reaction pathway was described in the presence (blue) or absence (red) of [EMIM]^+^. Top view of intermediate state geometries for the activation of CO_2_ (**c**) and adsorption of COOH (**d**) on Ag(111) surface in the presence of [EMIM]^+^. Ag atoms are represented in gray, C atoms in black, O atoms in red, N atoms in blue and H in white.

Figure 6 shows how the presence of the IL cation in the form ([EMIM]^+^ or [BMIM]^+^) helps to stabilize the activation of the CO_2_ at the interface of the Ag(111) electrode and how it helps to decrease the overpotential not only of the first-rate determining step but also of all the reaction intermediates involved in the EC CO_2_RR to CO. Indeed, the CO_2_RR pathway from CO_2_ to CO via adsorbed CO_2_ on the electrode surface (*CO_2_) is reported. When [EMIM]^+^ is explicitly considered in the simulation, the Gibbs energy variation follows the blue line in the reaction paths in Fig. 6a, b. All reaction steps are downhill when an external potential (E) of −1.67 V vs. Ag/AgCl is applied (left panel), in agreement with the experimental results exhibiting the CO_2_ conversion to CO. At the same applied potential, in the absence of the IL (red line), the crucial intermediate *CO_2_ is still uphill with a Gibbs energy variation of about +0.25 eV. This step reaches the equilibrium (right panel) only at a higher applied potential, E = −2.12 V vs. Ag/AgCl. This clearly demonstrates the role of the IL in promoting the *CO_2_ formation, thus lowering the onset potential of the RDS of the CO_2_RR, which is the formation and stabilization of the *CO_2_ species. Indeed, electrostatic interactions (non-covalent bonds) are formed between the *CO_2_ adsorbed on the Ag surface and IL cation, as shown in previous literature works^65,66^.

The CO_2_RR occurs on undercoordinated Ag atoms at (111) surface, i.e. the active sites at the electrode surface. By negatively charging the electrode, [BMIM]^+^ or [EMIM]^+^ are attracted to the surface and form a sort of imidazolium cation monolayer around the working electrode. The source of electrons, necessary for the reaction is the electrode, while protons originate from the membrane or from additional proton sources, such as water or acetic acid, particularly in the case of [EMIM]/[BMIM][CO_2_CH_3_]. The larger availability of protons in the latter cases enhances HER by further reducing the active sites for CO_2_RR, already hindered by adsorbed carbenes.

In Fig. 6c, d the activation of CO_2_ to *CO_2_ and the protonation of the latter are shown, respectively. CO_2_ is chemisorbed on the electrode surface, which acts as an electron donor. The cation, electrostatically attracted to the electrode surface is located near the active site of the CO_2_RR and stabilizes its intermediates through hydrogen bonds. We found that the cation is positioned parallel to the flat surface of the electrode to maximize interactions with both the Ag and CO_2_. Instead, [EMIM]^+^ or [BMIM]^+^ were not found to form any bond with Ag, contrary to what has been demonstrated with EMIM: and BMIM: that bond to Ag as illustrated Fig. 2-right, which explains the lowest selectivity for the CO_2_RR of the [CO_2_CH_3_]-based ILs in comparison to the [SO_3_CF_3_]-based ILs.

### [BMIM][SO_3_CF_3_] stability

**Fig. 7:**
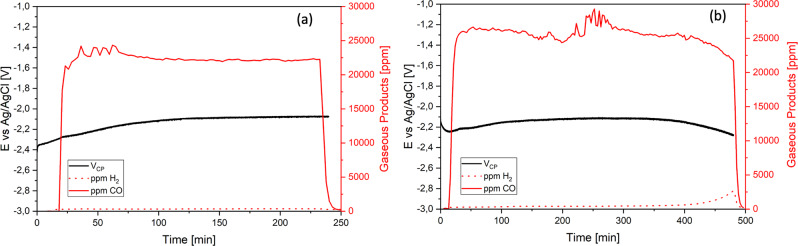
Stability test of [BMIM][SO_3_CF_3_] electrolyte during the electrocatalytic CO_2_ reduction reaction on Ag electrode. **a** CP for 8 h at −20 mA with 0.3 M [BMIM][SO_3_CF_3_] in ACN. **b** CP for 8 h at −60 mA with 0.3 M [BMIM][SO_3_CF_3_] in ACN. Black line represents the trend of potential at the fixed current value. Continuous and dotted red lines correspond to the CO and H_2_ concentrations in the outlet gas, respectively. Set-up: H-cell; WE: Ag foil; catholyte: 0.3 M IL in ACN; CE: Ni mesh; anolyte: 0.1 M KOH.

From the previous comparison between all six ionic liquids, it emerged that the best performances are those of [BMIM][SO_3_CF_3_], especially in terms of high selectivity towards the CO_2_ to CO conversion (see results of CPs in 3). For this reason, we have selected this IL for further investigations towards its practical application. Its stability and selectivity towards the EC CO_2_RR was proved in two consecutive long-term tests of 8 h, at two different applied currents. In Fig. 7a, the first 8 h CP at −20 mA is reported. A stable CO production (FE_CO_ = 94%) is observed throughout the time, while the production of H_2_ is close to zero (FE_H2_ = 0.5%). To evaluate eventual performance variations at higher and more relevant current values, a subsequent CP for additional 8 h at I = −60 mA was performed and the results are shown in Fig. 7b. A constant production of CO (FE_CO_ = 95%) was also measured at this higher current value and the HER was maintained close to zero (FE_H2_ = 0.3%). A good stability of the potential was maintained along the entire duration of both tests, as can be seen from the black lines in Fig. 7a, b. It can be concluded that [BMIM][SO_3_CF_3_] shows on average a constant potential, high production of CO (FE > 95%), almost zero production of H_2_ till a total of 8 h of continuous operation at relevant current values of up to −60 mA.

## Conclusions

Seven imidazolium salts were tested for the electrocatalytic CO_2_ conversion to CO diluted in acetonitrile to reduce their viscosity. Acetonitrile was chosen as solvent thanks to its ability to solubilize ILs and its lower molecular weight than most organic solvents, which guarantees high conductivity and low viscosity. It was demonstrated that the anionic part of imidazolium salts influences the CO_2_ solubility and the final conductivity of the solution, depending on fluorination degree of the anion. In particular, the type of anion regulates the ratio between cations and carbenes. Theoretically, it was demonstrated that the active species to work as co-catalyst in CO_2_RR is the cation. Instead, the carbene species can bind the electrode surface Ag(111) and decrease active sites for the EC CO_2_RR. It was found that imidazolium salts of acetate are more selective towards the production of H_2_ because of the anion role, while under the same conditions, imidazolium salts of triflate show a very high selectivity towards the production of CO (FE_CO_ > 95%), which is even better than the most employed imidazolium salts of tetrafluoroborate. The [BMIM][SO_3_CF_3_]-based electrolyte also shown a promising stability for a total of 16 h under relevant current values (I = −20 and −60 mA).

Our experimental and theoretical results proved that all the studied ILs lower the onset potential of the electrochemical CO_2_ reduction reaction, compared to the performance of the pure organic solvent. However, the selectivity towards the CO production instead of the H_2_ evolution depends on the IL anion. Future developments will concern the analysis of other ILs, different solvents, electrochemical cell configurations and a more in-depth study on the role of the ionic mobility of the cation and anion at different IL concentrations in the electrolyte solutions.

## Experimental methods

### Chemicals

The electrochemical studies were performed by using ionic liquids made of imidazolium salts because of their higher affinity for CO_2_. All the ILs listed in Table 1 were synthesized and supplied by IOLITEC GmbH (high purity grade) and used without any further purification.

The purity for the triflates was above 99%, measured by ion chromatography, while for the alkanoates >98% of purity was supplied. The water content of ionic liquid with acetate anion is in the range of 0.6–0.8%. For the triflate-containing ILs it is below 1000 ppm.

### Electrochemical cell and set-up

The EC CO_2_RR tests with each IL-based electrolyte were done in a two-compartment H-type cell (see set-up scheme, Supplementary Fig. S1, SI). The cell used for the experiment and all other glassware were cleaned with Milli-Q water (*R* > 18.2 MΩ, 25 °C) by several rinsing cycles in an ultrasound bath and dried under nitrogen stream.

The cathodic chamber contained the working electrode (WE: Ag Foil) and the reference electrode (RE: Ag/AgCl in saturated KCl), which were immersed in a solution of the ionic liquid in an organic solvent. Due to their high viscosity, ionic liquids are often mixed with organic solvents or water^67^. Because of the low solubility of CO_2_ and the competitive HER, a aqueous solutions is usually avoided. Acetonitrile (ACN) is highly suitable among the organic solvents found in literature since, in addition to increase the conductivity and decrease the viscosity of imidazolium salts, it is commonly used for the CO_2_ electro-reduction because the CO_2_ solubility in it is eight times greater than in water and it is electrochemically stable^68^. Therefore, a solution of 0.3 M IL in ACN was used for the EC CO_2_RR tests.

In the anodic chamber, a nickel mesh was used as counter electrode (CE), immersed in an aqueous solution of a strong basic electrolyte to promote a stable oxygen evolution reaction (OER). Potassium hydroxide (0.1 M KOH) was chosen because of its high conductivity, which also affects the total ohmic drops of the system and enhances the effective passage of current^69,70^. However, to avoid the transport of K^+^ ions from the anodic chamber to the cathodic one through the employed membrane, the use of a more concentrated KOH solution (e.g. 1 M) with a higher conductivity is not recommended since it was verified that a high concentration of K^+^ in the catholyte promotes the formation of a precipitate in the IL-based electrolyte. The salt formed was collected, dried and further analysed by ion chromatography (IC) and compared to original samples prepared separately to confirm that it is composed by a salt with a potassium cation and the anion of the IL under use, confirming migration through the membrane (see Fig. S2, SI).

The cell chambers were separated by a commercial bipolar membrane (BM, Fumasep FBM by Fumatech). The BM consists of an anion exchange layer (AEL), and a cation exchange layer (CEL) that were installed facing the anode and cathode compartments of the cell, respectively. At the interface between the AEL and CEL, water dissociates into OH^-^ and H^+^ ions. The membrane was stored in a saturated solution of sodium chloride and before its use it was activated with a solution of H_2_SO_4_ 0.1 M (CEL side) and KOH 0.1 M (AEL side) already assembled in the H-cell. This configuration prevented the mixing of the chambers content and avoids further oxidation of the products formed during the CO_2_ reduction. Further information regarding the ions transport mechanism through the bipolar membrane is illustrated in the SI, Fig. S3.

### Electrocatalytic CO_2_RR tests methods

The cell was equipped with a gas inlet/outlet, enabling the air purging with N_2_ and then with CO_2_. The N_2_ and CO_2_ stored in cylinders were sent to the cathodic compartment at a constant rate (20 mL/min), defined by a mass flow controller (Bronkhorst EL-FLOW), and bubbled directly in the electrolyte solution that was magnetically stirred to facilitate the CO_2_ dissolution and mass transport towards the cathode. Before the electrochemical tests, the solution was outgassed with N_2_ and then saturated with CO_2_. The electrochemical measurements were performed with a VoltaLab PGZ 301 Dynamic Potensiostat equipped with VoltaMaster 4 Software. The gas outlet of the cathodic compartment passes through a Genie filter to remove the moisture and then it was analyzed with a Varian 490 Micro GC equipped with a PPQ and a Molsieve columns. This allows the online analysis of the gaseous products generated during electrochemical tests.

Potentiodynamic electrochemical measurements were carried out to study the electrochemical stability window of each ionic liquid. Cyclic voltammetries (CVs) in N_2_ were performed in a variable potential window up to reach 1 mA cm^−2^ of anodic and cathodic currents.

For the electrocatalytic tests, CVs in N_2_ and CO_2_, linear sweep voltammetries (LSVs) and a chronopotentiometry (CP) were performed to evaluate the EC CO_2_ reduction in the imidazolium-based electrolytes saturated with CO_2_ at −20 mA for 2 h. During the CP, gaseous products were online analyzed with the Micro GC. The stability of the most performing IL was also evaluated under relevant applicative conditions through two CPs at −20 mA and −60 mA for 8 hours of continuous operation.

### Electrical conductivity of ILs

The electrical conductivity of each IL solution was measured with a conductivity meter with a two electrodes sensor (PC 80+ DHS by XS Instruments). The probe has a cell constant of 1 cm. A minimum volume of 5 mL of the pure ionic liquid, or its solution in ACN, were required to guarantee the full immersion of the probe in the liquid media into a glass vial of 20 mL. After each measurement, the probe was rinsed several times with Milli-Q water and dried with paper to avoid water contamination in subsequent solutions, which could alter the conductivity value.

### Viscosity of ILs

Viscosity measurements were performed with an Anton Paar Physica MCR 302 rotational rheometer. A 50 mm diameter parallel plate mode was used, and the gap between the two plates was set to 0.2 mm. A constant temperature of 25 °C was fixed. The viscosity of the pure ionic liquids and their solutions in ACN were measured with a rotation shear ramp ranging from 10 1/s to 10000 1/s.

### Field Emission Scanning Electron Microscopy (FESEM) and Energy Dispersive X-Ray spectroscopy (EDX)

A ZEISS Supra 40 Field Emission Scanning Electron Microscopy (FESEM) was employed to characterize the morphology of the Ag electrode. To quantify the elemental composition of the sample, Energy Dispersive X-Ray spectroscopy (EDX) characterization was performed via an in-house equipment (Oxford EDS microanalysis through a Si(Li) detector cooled with Liquid-N_2_).

### Raman spectroscopy

Raman spectra were obtained at room temperature (298 K) using a Horiba LabRAM HR Evolution spectrometer and a fiber-optic SuperHead Raman probe. The instrument is equipped with a multicanal CCD UV (1024 × 256 pixels) detector and 785 nm laser was used as excitation source to deliver a laser power of 100 mW. The electrode samples were measured with a 50x UV LWD confocal volume Olympus objective, 1 s of acquisition time to get 30 spectra for each sample. The liquid samples were collected in disposable plastic containers fit inside the superhead probe sample holder and cover with aluminum foil to prevent interference of the ambient light during the Raman measurements.

### X-ray photoelectron spectroscopy (XPS)

X-ray photoelectron spectroscopy (XPS) was carried out by using a PHI 5000 VersaProbe (Physical Electronics) system. The X-ray source was a monochromatic Al Kα radiation (1486.6 eV). Spectra were analyzed using Multipak 9.7 software. All core-level peak energies were referenced to C1s peak at 284.8 eV (C-C/C-H) and the background contribution in high resolution (HR) scans was subtracted by means of a Shirley function. For liquid analysis, an ionic liquid droplet has been directly positioned on a conductive double-sided tape on the sample holder and left in the loading chamber overnight, in order to let the volatile species to be pumped-out from the system by a turbomolecular pump. After required time the sample did not show any modification during the loading in the main chamber or during X-rays irradiation. The pressure inside the main chamber remained below 10^−5 ^Pa during the entire measurement.

### Computational details

DFT calculations were performed with the Quantum ESPRESSO code^71^. Electronic wavefunctions were expanded in plane-waves (PW), the exchange and correlation effects were modeled by the Perdew−Burke−Ernzerhof (PBE) functional^72^ and the electron-ion interaction was described by optimized ultrasoft pseudopotentials^73^. In all calculations a PW energy cutoff of 32 Ry (320 Ry) for the wavefunctions (electron densities) was adopted. All structures were relaxed by minimizing the atomic forces; convergence was assumed when the maximum component of the residual forces on the ions was smaller than 10^−5^ Ry/bohr. The Brillouin zone of the bulk Ag unit cell was sampled using a 10 × 10 × 10 shifted Monkhorst-Pack mesh and accordingly reduced in supercell calculations, to keep a constant k-point density. The calculated Ag lattice parameter resulted in 4.154 Å, in good agreement with previous DFT results^74^ and experimental values^75^. Surface calculations were performed with orthorhombic supercells containing slabs of four atomic layers where the atomic positions of the bottom surface layer were kept fixed to bulk positions during geometry optimizations. To avoid spurious interactions between periodic replicas, a vacuum region of at least 10 Å was added in the direction perpendicular to the surface.

The thermodynamic study of the electrochemical reactions was carried out within the grand canonical formulation proposed in^76^. By using a self-consistent continuum solvation model (SCCS), provided by the ENVIRON module^77^, and charged simulation cells, this method extends the Computational Hydrogen Electrode approach^78^, allowing to obtain the Gibbs free energy changes, at fixed electrode potential, also of reaction steps in which a different number of electrons and protons is exchanged. The chemical potentials of gas species (H_2_, CO_2_ and CO) were obtained following standard ideal-gas methods, considering 1 atm partial pressure, and T = 298 K. For the calculation of the enthalpic and entropic contributions to the Gibbs energy of the adsorbates, all degrees of freedom have been assumed as vibrational and treated in the harmonic approximation. To correct the systematic error introduced by the PBE functional in the energetics of the C = O bonds, following the procedure reported in ref. ^79,80^ we obtained corrections of −0.36 eV and +0.07 eV to the electronic energies of the CO and CO_2_ molecules, respectively.

Because of the low polarity, solvation effects due to the ACN solvent were treated implicitly through the SCCS by specifying the solvent static permittivity and surface tension at standard conditions. The use of the grand canonical approach and the SCCS allowed to explicitly investigate the stabilization effect of the cations on the CO_2_RR intermediates, by introducing a cation molecule ([EMIM]^+^) in the simulation cell.

The search for possible transition states of the studied reactions and the calculation of the corresponding activation energy was performed by means of Nudged Elastic Band (NEB) calculations^81^.

## Supplementary information

Supplementary Information

## Data Availability

The authors declare that the data supporting the findings of this study are available within the paper and its Supplementary Information files. Should any raw data files be needed in another format they are available from the corresponding author upon reasonable request. All data relevant to the electronic structure calculations mentioned in the manuscript have been uploaded on the Materials Cloud repository and can be found at the link: https://archive.materialscloud.org/deposit/records/1713.
