# Supplementary material for: Understanding the role of imidazolium-based ionic liquids in the electrochemical CO2 reduction reaction
Source: Commun Chem. 2023 Apr 29;6:84. doi: 10.1038/s42004-023-00875-9 (PMC10148827; doi:10.1038/s42004-023-00875-9)
Supplement: Supplementary file 1 — Supplementary Information [file 42004_2023_875_MOESM1_ESM.pdf]

Supporting Information

Understanding the role of imidazolium-based ionic liquids in the electrochemical CO_2 reduction reaction

Alessia Fortunati[1], Francesca Risplendi[1,*], Michele Re Fiorentin[1], Giancarlo Cicero[1], Emmanuele Parisi[1], Micaela Castellino[1], Elena Simone[1], Boyan Iliev[2], Thomas J.S. Schubert[2], Nunzio Russo[1], Simelys Hernández[1, *]

[1] *Department of Applied Science and Technology, Politecnico di Torino, Corso Duca degli Abruzzi 24, 10129, Turin, Italy*
[2] *Iolitec Ionic Liquids Technologies GmbH, Im Zukunftspark 9, 74076 Heilbronn, German*

**Corresponding authors: simelys.hernadez@polito.it; francesca.risplendi@polito.it*

Table of Contents

Table S1 : Literature data of electrocatalytic CO_2 reduction in ionic liquids using silver as a working electrode. 4

Figure S1: Scheme of the set-up used for the electrocatalytic CO_2 reduction reaction with the ionic liquid (IL)-based electrolytes. 6

Figure S2: Schematic representation of the K^+ cations transfer from the anodic to the cathodic compartment of the electrochemical cell, occurring when using a high concentration of KOH in the anolyte (e.g. 1M). (Right) Picture of the cathodic chamber evidencing the formation of a precipitate in the IL-based catholyte. 7

Figure S3: Bipolar membrane mounting scheme in the cell and working mechanism. 7

Table S2: Experimental conditions of the cyclic voltammetry (CV) test used to measure the electrochemical stability window of the IL-based electrolytes. 7

Figure S4: Potential stability range (cathodic potential) of 0.3M solutions of the six ILs in ACN in N_2 saturated atmosphere, considering a cut-off current density of 1 mA/cm^2. (A) Cyclic voltammetries obtained after a screening of CVs done at intervals of increasing cathodic potential, starting from the interval [0; -100mV] and increasing the lower limit until the curve reached a current value in the Y axis equal to -1 mA/cm^2. That value of current, was chosen as threshold based on previous literature studies. (B) histogram with the resulting cathodic stability windows of the different IL solutions. Catholyte: 0.3M IL in ACN, WE: 3 cm^2 Ag foil, anolyte: 0.1M KOH; CE: Ni Mesh; membrane: bipolar membrane. 8

Figure S5: Fresh catholyte solution (A), tested catholyte solution (B) and H_2 evolution over time (C) measured with a micro-gas chromatograph (GC) during 2h of CP to investigate the [BMIM][SO_3CF_3]-electrolyte reduction in N_2 saturated atmosphere. (D) Potential variation during the CP(2h, 20 mA/cm^2). Catholyte: 0.3M [BMIM][SO_3CF_3] in can, WE: 3 cm^2 Ag foil, anolyte: 0.1M KOH; CE: Ni Mesh; membrane: bipolar membrane. 8

Figure S6: Fresh catholyte solution (A), tested catholyte solution (B) and H_2 evolution over time (C) measured with a micro-GC during 2h of CP to investigate the [BMIM][CO_2CH_3]-electrolyte reduction in N_2 saturated atmosphere. (D) Potential variation during CP(2h, 20 mA/cm^2). Catholyte: 0.3M [BMIM][CO_2CH_3] in ACN, WE: 3 cm^2 Ag foil; anolyte: 0.1M KOH, CE: Ni Mesh, membrane: bipolar membrane. 9

Figure S7: XRD spectra of Ag foil used as working electrode evidencing the predominant crystalline face: Ag(111), based on the card reference JCPDS No.: 00-004-0783. 9

Figure S8: NEB reaction path for BMIM:-CO_2, EMIM:-CO_2 and EMIM:-Ag(111) reactions. The absence of a transition state shows that these reactions are barrierless. 10

Figure S9: Cyclic voltammetry curves (CVs) of [EMIM][CO_2CH_3] (A), [BMIM][CO_2CH_3] (B), [BMIM][CO_2CF_3] (C), [BMIM][BF_4] (D), [EMIM][SO_3CF_3] (E) and [BMIM][SO_3CF_3] (F) solutions [IL]=0.3M in ACN. Dashed and continuous lines represent CVs in N_2 and CO_2 saturated atmosphere, respectively. Catholyte: 0.3M IL in ACN, WE: 3 cm^2 Ag foil; anolyte: 0.1M KOH, CE: Ni Mesh, membrane: bipolar membrane. 10

Figure S10: Linear sweep voltammetry (LSV) curves of BMIM-containing ILs (A) and EMIM-containing ILs (B) solutions [IL]=0.3M in ACN. Black line is the blank test in pure ACN without IL. 11

Figure S11: Chronoamperometries curves registered during 2h test at J=-20 mA/cm^2. Catholyte: [ILs]=0.3M in ACN, WE: 3 cm^2 Ag foil; anolyte: 0.1M KOH, CE: Ni Mesh, membrane: bipolar membrane. 11

Figure S12: Raman spectra showing (A) a comparison between pure ACN (blue line), pure [BMIM][CO_2CH_3] (green line) and the mixed final electrolyte and (B) a comparison between the fresh electrode (dark green line) and the cleaned electrode after test (black line) (B). 12

Figure S13: FESEM images of the fresh Ag electrode: (A) before the test, (B) after the electrochemical tests in 0.3M [BMIM][SO_3CF_3] solution in ACN, including CVs, LSVs and CP at -20 mA for 2h, and (C) after the

electrochemical test and cleaning of the electrode by rinsing it with pure ACN. EDX analysis are reported in Table S3 12

Table S3: EDX data of Ag electrode just after the electrochemical tests (Figure S13-B), including CVs, LSVs and CP at -20 mA for 2h, in comparison with the Ag electrode after testing and cleaning it with ACN Figure S 13-C)........ 12

Figure S15: Raman spectra showing a comparison between Ag foil after test in [1-Butyl-2,3-Methyl imidazole acetate]=0.3M in ACN (purple line) and the same electrode cleaned by rinsing it with acetonitrile (black line). In addition to the peaks assigned and reported in Table 2, the post-test electrode spectrum shows peaks at 2925 and 2865 cm^{-1}, which are characteristic of the -CH_3 group attached to a ring. 14

Table S1 : *Literature data of electrocatalytic CO_2 reduction in ionic liquids using silver as a working electrode.*

Electrode	Product (Faradaic efficiency, %)	Electrolyte	Current density, mA/cm^2	Reference
Ag	Dimethyl carbonate (74)	Bare [BMIM][BF_4]	charge passed, 1.0 $F.mol^{-1}$	Zhang et al., 2008
Ag	CO (96)	18% [EMIM][BF_4] in water	n/a	Rosen et al., 2011
Ag	HCO^{-}_2 (95)	[P66614][124Triz] in Acetonitrile + water	Charge (10 C)	Hollingsworth et al., 2015
Ag	HCO^{-}_2 (6) CO (6)<br>H_2 (41)	0.1 mol dm^{-3} [P66614][124Triz] in ACN + 0.7 mol dm^{-3} of water	Charge (10 C)	Hollingsworth et al., 2015
Ag	n/a	Bare [PMIM][NTf_2]	−0.7	Tanner et al., 2016
Ag	n/a	Bare [EMIM][NTf_2]	−1.5	Tanner et al., 2016
Ag	n/a	Bare [BMIM][NTf_2]	−1.60	Tanner et al., 2016
Ag	n/a	Bare [BMIM][BF_4]	−0.8	Tanner et al., 2016
Ag	n/a	0.1 M [Bu_4 N][PF_6] + 0.02 M [Ethyl 2-Methyl Imidazolium][BF_4]	−10.0	Lau et al., 2016
Ag	n/a	0.1 M [Bu_4 N][PF_6] + 0.02 M [Ethyl 2,3-dimethyl Imimidazolium][BF_4]	−16.0	Lau et al., 2016
Ag	n/a	0.1 M [Bu_4 N][PF_6] + 0.02 M [Ethyl 2,3,4,5-tetramethyl Imimidazolium][BF_4]	−5.5	Lau et al., 2016
Ag	CO (90)	[Bmim][CF_3 SO_3]/<br>Propylene carbonate	−4.6	Shi et al., 2014

Ag	Formate (95)	0.1 M [P66614][124Triz] /Acetonitrile	n/a	Hollingsworth et al., 2015
Ag	CO (95.8)	[BMIm][Cl] in ethylene glycol (1:2)	n/a	Vasilyev, D.V et al., 2019
Ag	CO	75 mM H_2O/[Emim]BF_4	−4	Salehi-Khojin et al., 2013
Ag	HCOOH (63)	0.1 mol L^1 [P66614] 124Triz/acetonitrile	n/a	Hollingsworth et al., 2015
Ag	CO (100)	0.02 M 1,3-dimethyl-2-phenyl-imidazolium tetrafluoroborate/0.1 M $TBAPF_6$/7 mL acetonitrile	−4.2	Lau et al., 2016
Ag	CO (95.6+/-6-.8)	50 mol % [Emim]TFO+ $KHCO_3/H_2O$	−10	Neubauer et al., 2016
Ag	CO(70.4)	0.1 M n-Bu_4NPF_6/ 2.0 mM [C_{10}mim]BF_4 + 1.0% H_2O/acetonitrile	n/a	Zhao et al., 2016
Ag	CO (90)	[Bmim]BF_4/H_2O (<70%)	−1	Rudnev et al., 2017
Ag	CO (94)	[Bmim]BF_4/20% H_2O	n/a	Rudnev et al., 2017
Ag	CO (100)	1 M [EmimOH]Cl/2 M Ethylene Glycol/Propylene carbonate	−4	Vasilyev et al., 2019
Ag	CO (80) H_2 (17)	[BMIM][BF_4]=0.3M in Acetonitrile	−20	This work
Ag	CO (75) H_2 (10)	[BMIM][CO_2CF_3]=0.3M in Acetonitrile	−20	This work
Ag	CO (20) H_2 (80)	[BMIM][CO_2CH_3]=0.3M in Acetonitrile	−20	This work
Ag	CO (90) H_2 (5)	[EMIM][CO_2CH_3]=0.3M in Acetonitrile	−20	This work
Ag	CO (97) H_2 (2)	[BMIM][SO_3CF_3]=0.3M in Acetonitrile	−20	This work
Ag	CO (95) H_2 (3)	[EMIM][SO_3CF_3]=0.3M in Acetonitrile	−20	This work
Ag	CO (93) H_2 (2.4)	[BMIM][BF_4]=0.3M in Acetonitrile	−10	This work
Ag	CO (96) H_2 (3.2)	[BMIM][CO_2CF_3]=0.3M in Acetonitrile	−10	This work

Ag	CO (12)<br>H_2 (85)	[BMIM][CO_2CH_3]=0.3M in Acetonitrile	−10	This work
Ag	CO (10)<br>H_2 (85)	[EMIM][CO_2CH_3]=0.3M in Acetonitrile	−10	This work
Ag	CO (95)<br>H_2 (0.3)	[BMIM][SO_3CF_3]=0.3M in Acetonitrile	−10	This work
Ag	CO (85)<br>H_2 (1.5)	[EMIM][SO_3CF_3]=0.3M in Acetonitrile	−10	This work
Ag	CO (92)<br>H_2 (6)	[BMIM][BF_4]=0.3M in Acetonitrile	−5	This work
Ag	CO (98)<br>H_2 (0.4)	[BMIM][CO_2CF_3]=0.3M in Acetonitrile	−5	This work
Ag	CO (70)<br>H_2 (20)	[BMIM][CO_2CH_3]=0.3M in Acetonitrile	−5	This work
Ag	CO (17)<br>H_2 (75)	[EMIM][CO_2CH_3]=0.3M in Acetonitrile	−5	This work
Ag	CO (98)<br>H_2 (0.7)	[BMIM][SO_3CF_3]=0.3M in Acetonitrile	−5	This work
Ag	CO (89)<br>H_2 (1.9)	[EMIM][SO_3CF_3]=0.3M in Acetonitrile	−5	This work

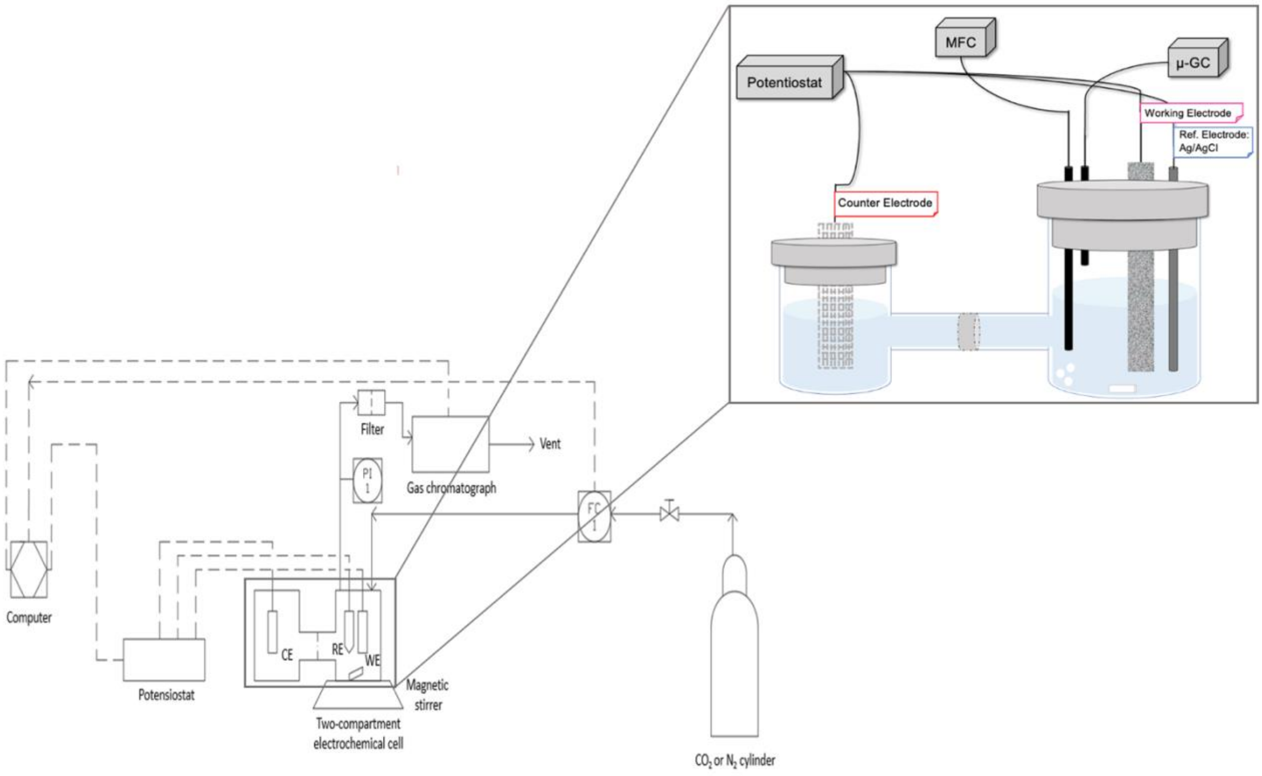

Figure S1: *Scheme of the set-up used for the electrocatalytic CO_2 reduction reaction with the ionic liquid (IL)-based electrolytes.*

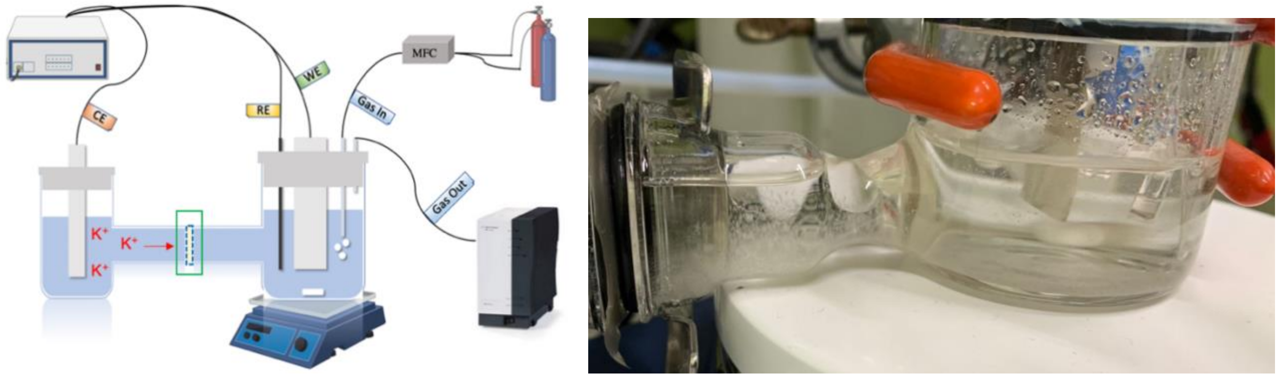

Figure S2: *Schematic representation of the K^+ cations transfer from the anodic to the cathodic compartment of the electrochemical cell, occurring when using a high concentration of KOH in the anolyte (e.g. 1M). (Right) Picture of the cathodic chamber evidencing the formation of a precipitate in the IL-based catholyte.*

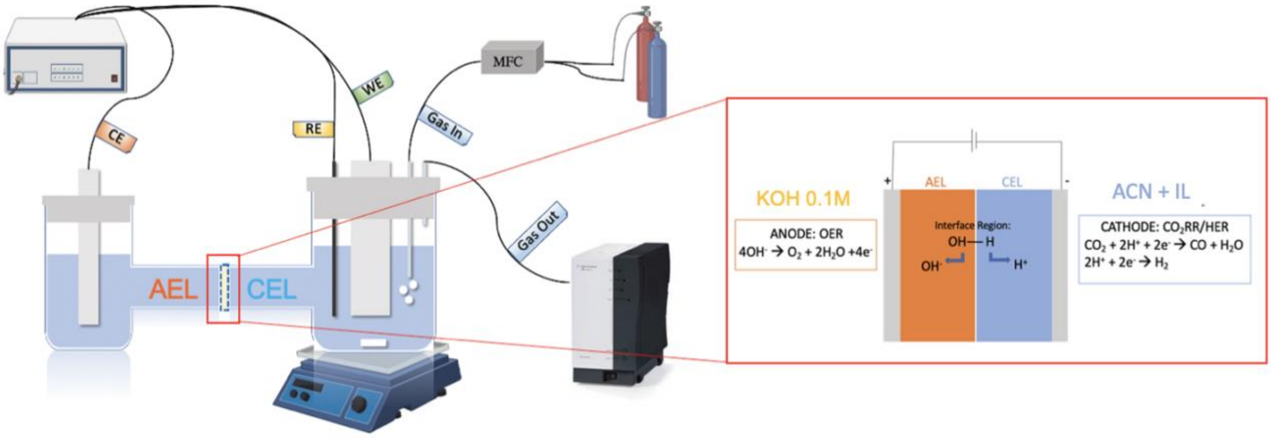

Figure S3: *Bipolar membrane mounting scheme in the cell and working mechanism.*

Table S2*: Experimental conditions of the cyclic voltammetry (CV) test used to measure the electrochemical stability window of the IL-based electrolytes.*

Parameter	Value
Temperature	298.15 K
Pressure	1 atm
Catholyte solution	0.3M IL (see Table 1) in acetonitrile (ACN)
Anolyte solution	0.1M KOH in H_2O
Scanning rate	30 mVs^{-1}
Working Electrode (WE)	Silver foil (3 cm^2)
Counter Electrode (CE)	Nickel mesh
Reference Electrode (RE)	Ag/AgCl in sat. KCl

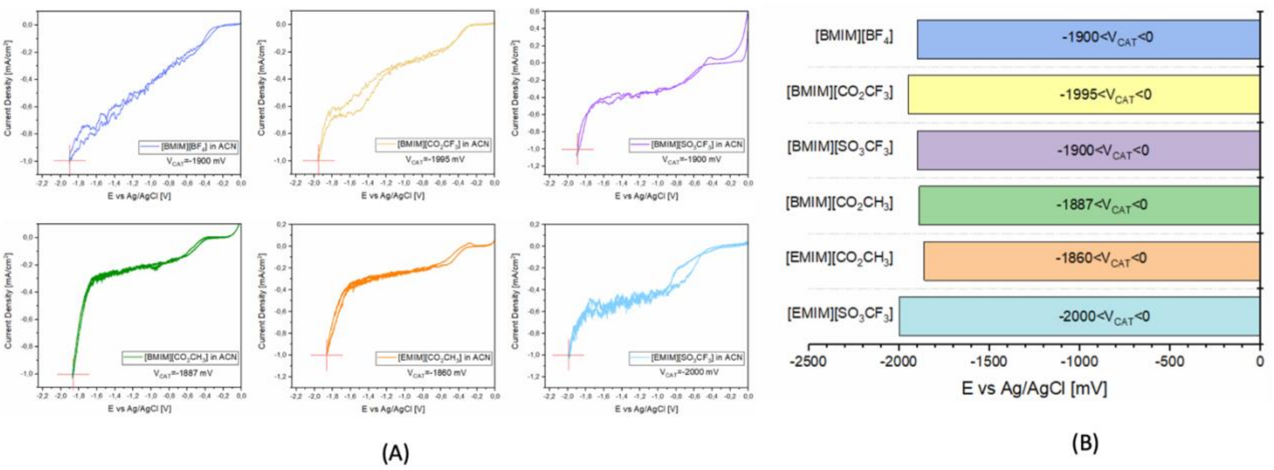

Figure S4: *Potential stability range (cathodic potential) of 0.3M solutions of the six ILs in ACN in N_2 saturated atmosphere, considering a cut-off current density of 1 mA/cm^2. (A)* Cyclic *voltammetries* obtained after a screening of CVs done *at intervals of increasing* cathodic *potential, starting from the interval [0; -100mV] and increasing the lower limit until the curve reached a current value in the Y axis equal to -1 mA/cm^2. That value of current, was chosen as threshold based on previous literature studies. (B) histogram* with the resulting cathodic stability windows of the different IL solutions. *Catholyte: 0.3M IL in ACN, WE: 3 cm^2 Ag foil, anolyte: 0.1M KOH; CE: Ni Mesh; membrane: bipolar membrane.*

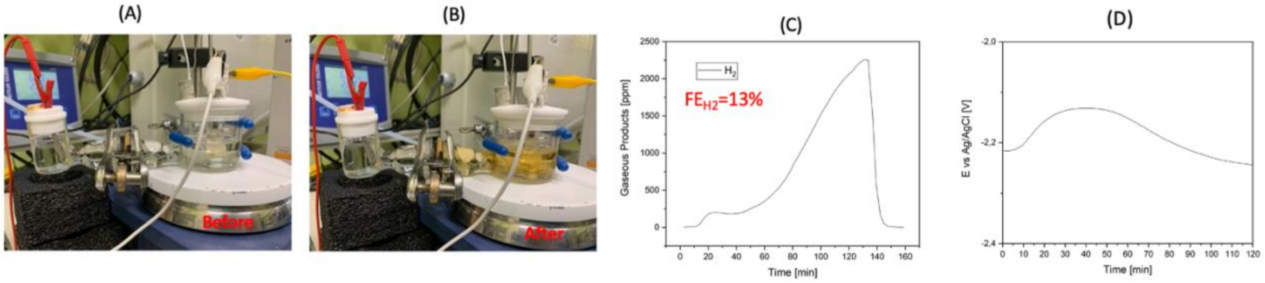

Figure S5: *Fresh catholyte solution (A), tested catholyte solution (B) and H_2 evolution over time (C) measured with a micro-gas chromatograph (GC) during 2h of CP to investigate the [BMIM][SO_3CF_3]-electrolyte reduction in N_2 saturated atmosphere. (D) Potential variation during the CP(2h, 20 mA/cm^2). Catholyte: 0.3M [BMIM][SO_3CF_3] in can, WE: 3 cm^2 Ag foil, anolyte: 0.1M KOH; CE: Ni Mesh; membrane: bipolar membrane.*

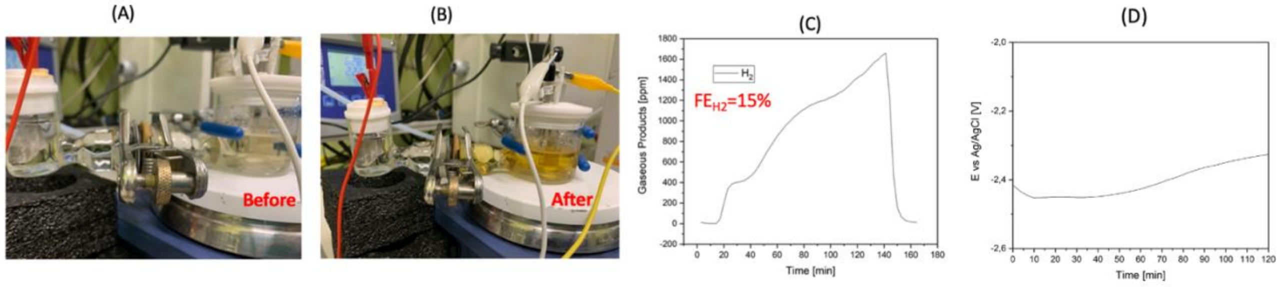

Figure S6: *Fresh catholyte solution (A), tested catholyte solution (B) and H_2 evolution over time (C) measured with a micro-GC during 2h of CP to investigate the [BMIM][CO_2CH_3]-electrolyte reduction in N_2 saturated atmosphere. (D) Potential variation during CP(2h, 20 mA/cm^2). Catholyte: 0.3M [BMIM][CO_2CH_3] in ACN, WE: 3 cm^2 Ag foil; anolyte: 0.1M KOH, CE: Ni Mesh, membrane: bipolar membrane.*

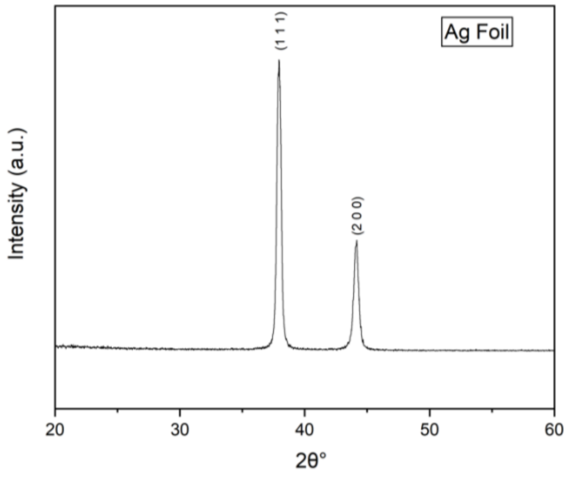

Figure S7*: XRD spectra of Ag foil used as working electrode evidencing the predominant crystalline face: Ag(111), based on the card reference JCPDS No.: 00-004-0783.*

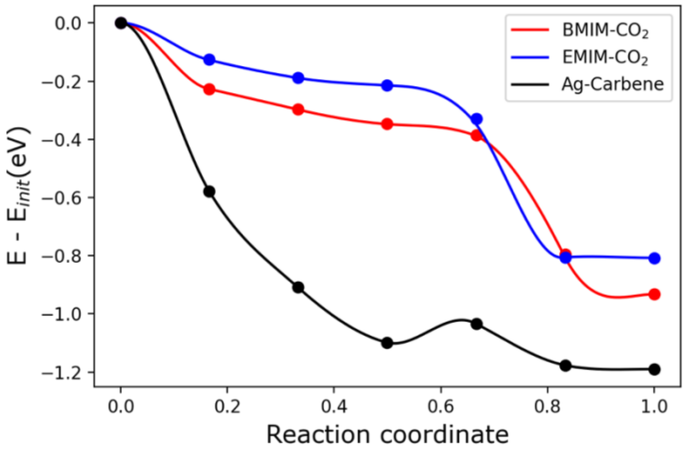

Figure S8: *NEB reaction path for BMIM:-CO_2, EMIM:-CO_2 and EMIM:-Ag(111) reactions. The absence of a transition state shows that these reactions are barrierless.*

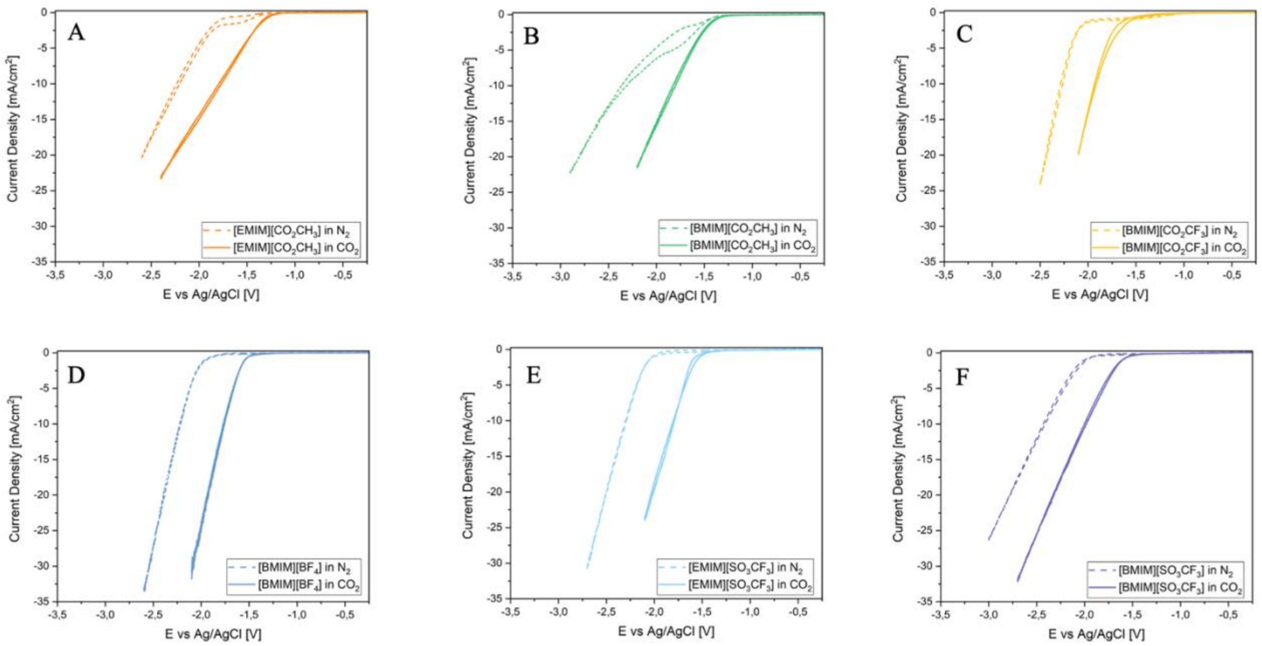

Figure S9: *Cyclic voltammetry curves (CVs) of [EMIM][CO_2CH_3] (A), [BMIM][CO_2CH_3] (B), [BMIM][CO_2CF_3] (C), [BMIM][BF_4] (D), [EMIM][SO_3CF_3] (E) and [BMIM][SO_3CF_3] (F) solutions [IL]=0.3M in ACN. Dashed and continuous lines represent CVs in N_2 and CO_2 saturated atmosphere, respectively. Catholyte: 0.3M IL in ACN, WE: 3 cm^2 Ag foil; anolyte: 0.1M KOH, CE: Ni Mesh, membrane: bipolar membrane.*

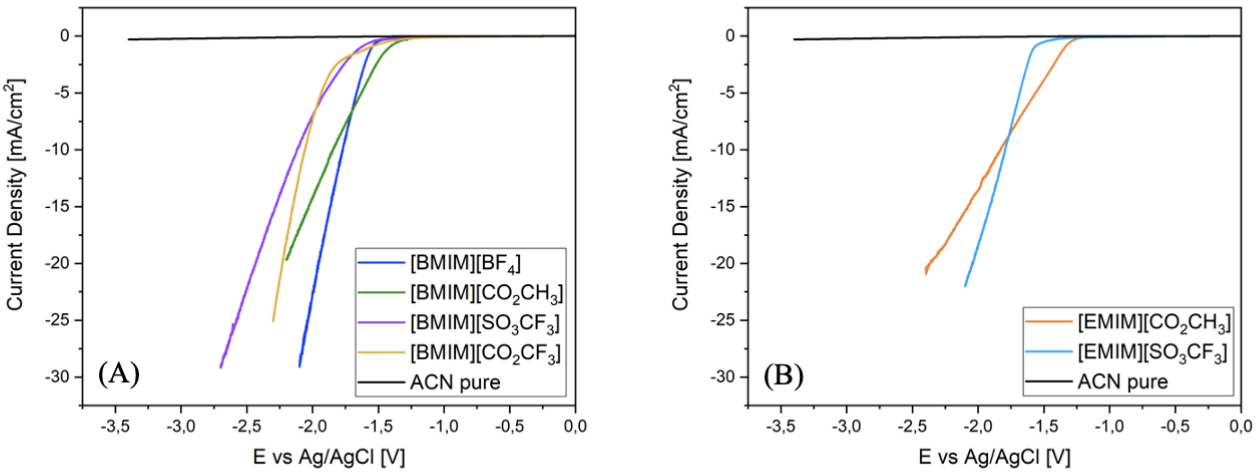

Figure S10: *Linear sweep voltammetry (LSV) curves of BMIM-containing ILs (A) and EMIM-containing ILs (B) solutions [IL]=0.3M in ACN. Black line is the blank test in pure ACN without IL.*

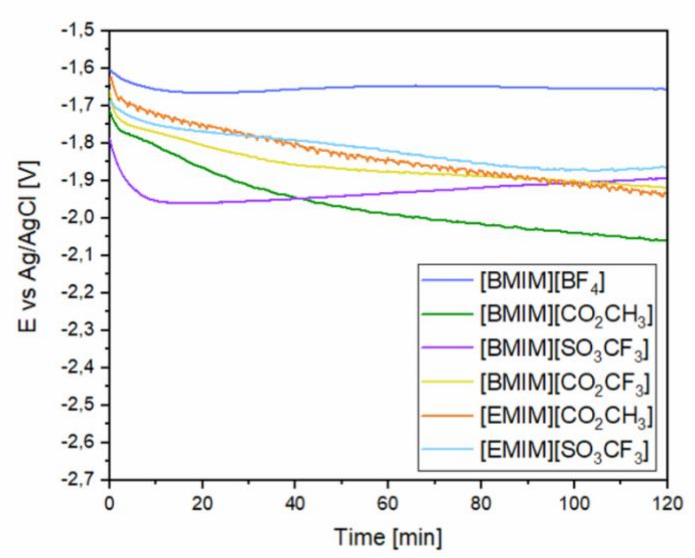

Figure S11: *Chronoamperometries curves registered during 2h test at J=-20 mA/cm^2. Catholyte: [ILs]=0.3M in ACN, WE: 3 cm^2 Ag foil; anolyte: 0.1M KOH, CE: Ni Mesh, membrane: bipolar membrane.*

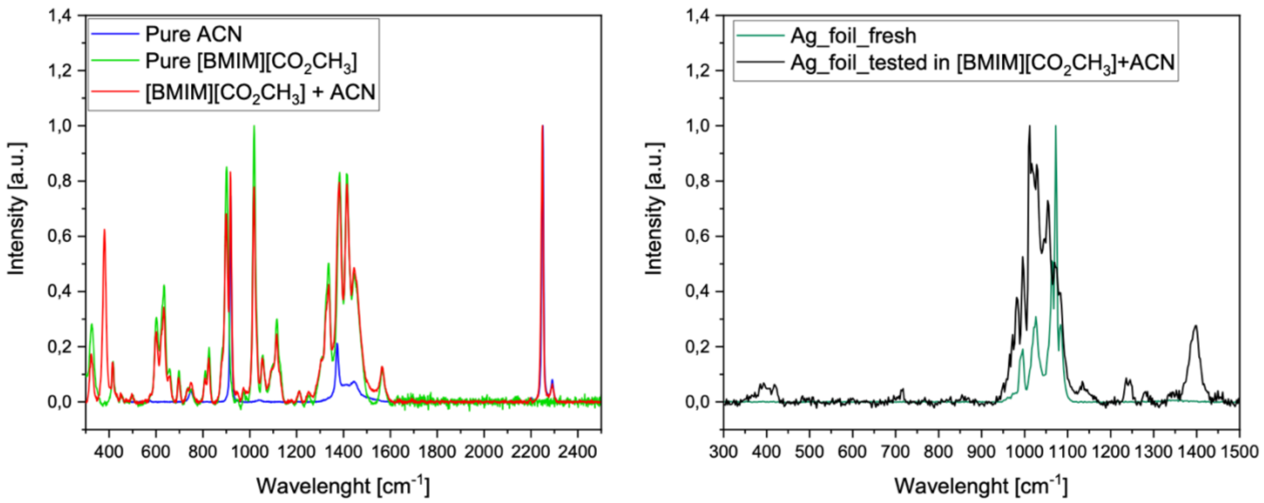

(A) (B)

Figure S12: *Raman spectra showing (A) a comparison between pure ACN (blue line), pure [BMIM][CO_2CH_3] (green line) and the mixed final electrolyte and (B) a comparison between the fresh electrode (dark green line) and the cleaned electrode after test (black line) (B).*

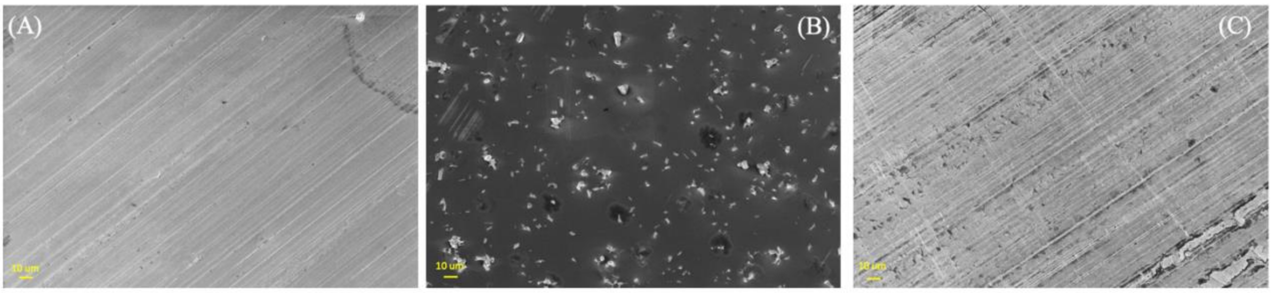

Figure S13**:** *FESEM images of the fresh Ag electrode: (A) before the test, (B) after the electrochemical tests in 0.3M [BMIM][SO_3CF_3] solution in ACN, including CVs, LSVs and CP at -20 mA for 2h, and (C) after the electrochemical test and cleaning of the electrode by rinsing it with pure ACN. EDX analysis are reported in* ***Table S3*** *.*

Table S3*: EDX data of Ag electrode just after the electrochemical tests (Figure S13-B), including CVs, LSVs and CP at -20 mA for 2h, in comparison with the Ag electrode after testing and cleaning it with ACN* ***Figure S13****-C).*

Elements	Ag_tested (Atomic%)	Ag_tested_cleaned (Atomic %)
C	23.56	0
O	32.22	0
F	24.88	0
S	2.90	0
K	2.16	0
Ag	14.28	100

The electrode was tested with [BMIM][SO_3CF_3] (Figure S13-B) and EDX analysis evidenced the presence of: S and F, characteristic elements of the anion; C and O belonged to the IL cation or to the solvent; Ag from electrode surface and K due to anolyte crossover through the membrane. To conclude, on electrode just tested and not cleaned the authors found the presence of the anion on the electrode surface. Then, the electrode after test and EDX analysis was rinsed with ACN, in order to remove any residue from the surface and analyze any pure surface reconstruction. EDX analysis performed on cleaned tested electrode (Table S3) did not show anion traces on electrode surface.

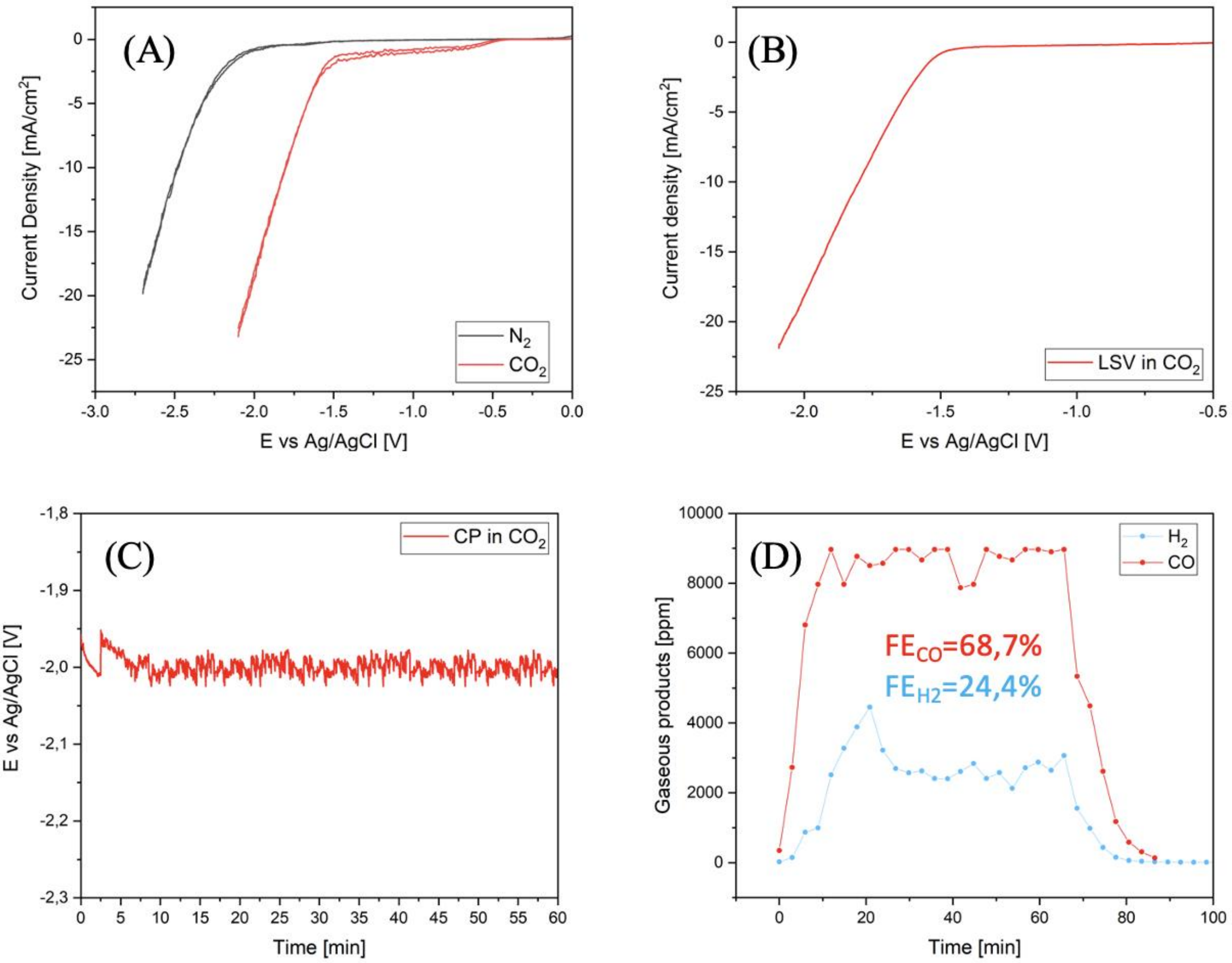

Figure S14: *Results of electrochemical test in an H-type cell with 1-Butyl-2,3-Methyl Imidazole acetate as electrolyte, [IL]=0.3M in ACN. (A) Cyclic voltammetry in N_2 and CO_2; (B) LSV in CO_2; (C) CP for 1h at I=-20mA in CO_2; (D) Gaseous products concentration measured by a micro-GC during the CP test.*

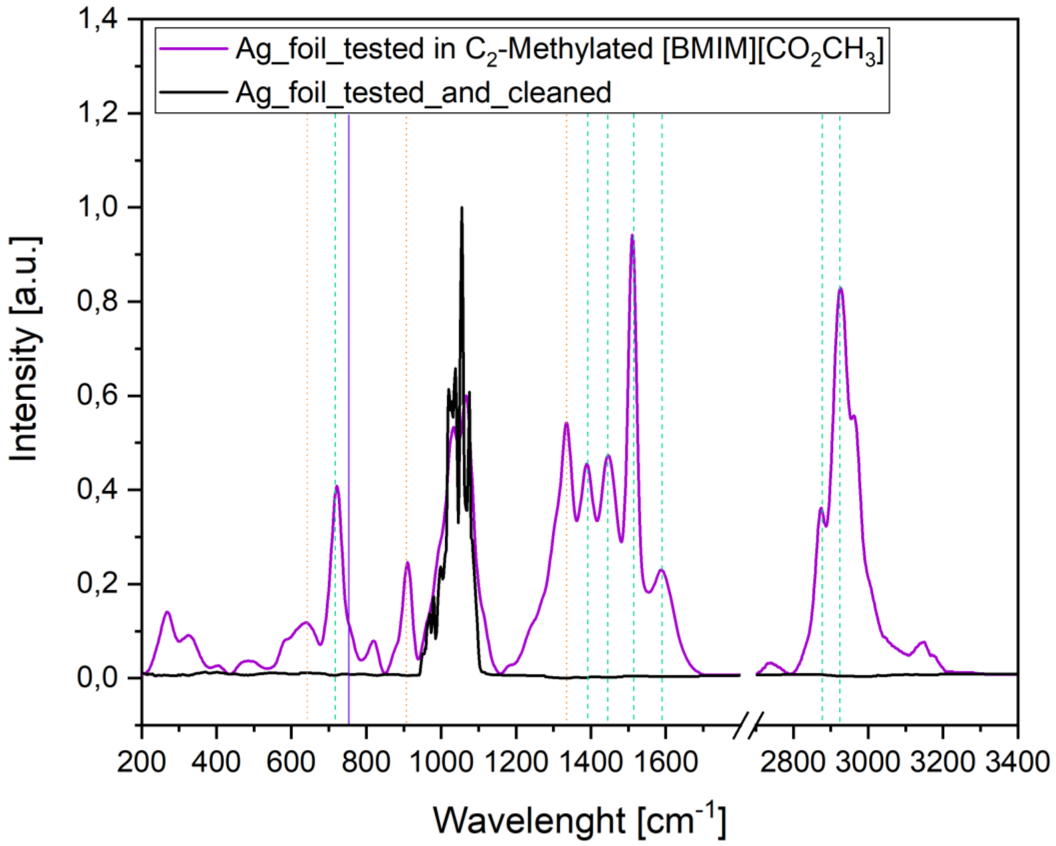

Figure S15: *Raman spectra showing a comparison between Ag foil after test in [1-Butyl-2,3-Methyl imidazole acetate]=0.3M in ACN (purple line) and the same electrode cleaned by rinsing it with acetonitrile (black line). In addition to the peaks assigned and reported in Table 2, the post-test electrode spectrum shows peaks at 2925 and 2865 cm*$^{-1}$*, which are characteristic of the -*CH_3 *group attached to a ring. Vertical lines mark intensity peaks related to acetate anion (dot orange line), BMIM cation (dashed green line) and finally ACN (solid purple line).*
